# Rods progressively escape saturation to drive visual responses in daylight conditions

**DOI:** 10.1038/s41467-017-01816-6

**Published:** 2017-11-27

**Authors:** Alexandra Tikidji-Hamburyan, Katja Reinhard, Riccardo Storchi, Johannes Dietter, Hartwig Seitter, Katherine E. Davis, Saad Idrees, Marion Mutter, Lauren Walmsley, Robert A. Bedford, Marius Ueffing, Petri Ala-Laurila, Timothy M. Brown, Robert J. Lucas, Thomas A. Münch

**Affiliations:** 1 Retinal Circuits and Optogenetics, Centre for Integrative Neuroscience and Bernstein Center for Computational Neuroscience, University of Tübingen, 72076 Tübingen, Germany; 2 International Max Planck Research School, University of Tübingen, 72074 Tübingen, Germany; 3 Faculty of Biology Medicine and Health, University of Manchester, Manchester, M13 9PT UK; 4 Institute for Ophthalmic Research, Department of Ophthalmology, University of Tübingen, 72076 Tübingen, Germany; 5 Department of Biosciences, University of Helsinki, 00014 Helsinki, Finland; 6 Department of Neuroscience and Biomedical Engineering (NBE), Aalto University School of Science and Technology, 00076 Espoo, Finland; 7 Present Address: Department of Neurosurgery and Hansen Experimental Physics Laboratory, Stanford University, Stanford, California 94305-4085 USA; 8 Present Address: Visual Circuits Laboratory, Neuro-Electronics Research Flanders, IMEC, KU Leuven and VIB, 3001 Leuven, Belgium; 9 Present Address: Institute of Pharmacy, Department of Pharmacology and Toxicology, University of Innsbruck, A-6020 Innsbruck, Austria; 10 Present Address: Stryker Imorphics, Worthington House, Towers Business Park, Wilmslow Road, Manchester, M20 2HJ UK

## Abstract

Rod and cone photoreceptors support vision across large light intensity ranges. Rods, active under dim illumination, are thought to saturate at higher (photopic) irradiances. The extent of rod saturation is not well defined; some studies report rod activity well into the photopic range. Using electrophysiological recordings from retina and dorsal lateral geniculate nucleus of cone-deficient and visually intact mice, we describe stimulus and physiological factors that influence photopic rod-driven responses. We find that rod contrast sensitivity is initially strongly reduced at high irradiances, but progressively recovers to allow responses to moderate contrast stimuli. Surprisingly, rods recover faster at higher light levels. A model of rod phototransduction suggests that phototransduction gain adjustments and bleaching adaptation underlie rod recovery. Consistently, exogenous chromophore reduces rod responses at bright background. Thus, bleaching adaptation renders mouse rods responsive to modest contrast at any irradiance. Paradoxically, raising irradiance across the photopic range increases the robustness of rod responses.

## Abstract (web summary)

Rod photoreceptors are thought to be saturated under bright light. Here, the authors describe the physiological parameters that mediate response saturation of rod photoreceptors in mouse retina, and show that rods can drive visual responses in photopic conditions.

## Introduction

Vision functions over about a dozen decades of light intensity^1, 2^ thanks in part to the use of two different photoreceptor classes (rods and cones). Rods are specialized for high-fidelity signaling at low-light levels, whereas cones mediate fast signaling at higher light levels. Based on this division of labor, light intensities are called scotopic (only rods are active, starlight vision), mesopic (both rods and cones are active), or photopic (rods are saturated, and only cones are active, daylight vision).

The distinction between mesopic and photopic conditions is, by definition, determined by the background irradiance at which rods saturate. However, rod saturation is not as absolute as commonly presented. Rod saturation becomes apparent as a reduction in contrast sensitivity at higher irradiances^3–5^ (seen as deviation from Weber–Fechner law). As a result, larger contrasts still elicit responses from rods, but moderate contrast stimuli only trigger cone responses. At higher irradiances, this “incremental saturation” could progress sufficiently to produce a state of effective “absolute saturation”, in which rods are unresponsive to any physiological contrast. However, whether rods do indeed reach absolute saturation and, if so, at what irradiance this occurs, remain uncertain. Phenomena interpreted as rod saturation have been reported in ex vivo recordings at irradiances ranging from 10^2^ to 10^5^ rhodopsin isomerizations per rod per second (R* rod^–1^ s^–1^)^4, 6–8^. However, other studies reveal that rod responses can be recorded across these light intensities^9, 10^, and recent behavioral studies^11, 12^ provided evidence for functional rod vision at up to 10^5^ R* rod^–1^ s^–1^. Presumably, these discrepancies reflect methodological differences, e.g., species studied, end point measured, or experimental conditions^5, 13–15^. Overall, however, the capacity of rods to contribute to visual responses at higher irradiances remains incompletely understood.

We set out to explicitly define the limits of rod vision in mice by recording rod responses over a wide range of irradiances, using ex vivo and in vivo electrophysiological recordings. Surprisingly, we do not find a simple relationship between rod saturation and irradiance. While rod contrast sensitivity is impaired upon stepping to backgrounds of 10^4^ R* rod^–1^ s^–1^ and brighter, contrast sensitivity recovers over time under all backgrounds. In fact, we see rod responses to moderate contrast stimuli even at 10^7^ R* rod^–1^ s^–1^, the brightest background we tested. Moreover, contrast sensitivity recovers faster at higher irradiance, such that, contrary to conventional wisdom, raising the background light intensity within the “photopic” range does not reduce, but increase rod contribution to visual responses.

## Results

### Irradiance-dependent changes in rod contrast sensitivity

**Fig. 1:**
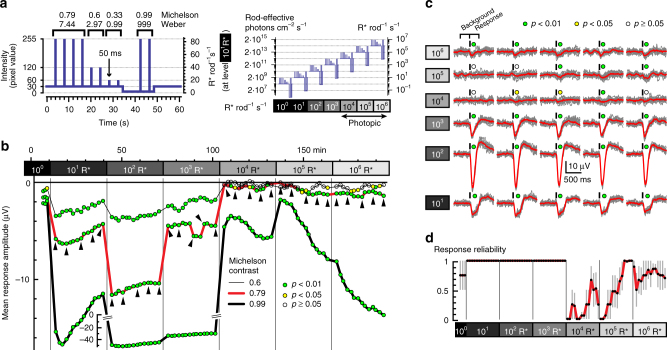
Ex vivo ERG recordings from isolated *Cnga3* ^*−/−*^ retina. **a** Stimulus used for ex vivo ERG recordings consisted of 50 ms flashes ranging from 0.33 to 0.99 Michelson contrast. Right: absolute stimulus intensities at different light levels. **b**, **c** Data from one representative retina. **b** Running average of the ERG response (baseline-corrected, i.e., mean negative voltage deflections in the 300 ms after flash onset, minus 300 ms before). Each data point shows mean from three consecutive stimuli (i.e., from 12 individual flashes for 0.79 Michelson contrast, and 6 flashes for the other contrasts; data for the weakest contrast is omitted for clarity). Neighboring data points are shifted by one stimulus. The color-coded disks indicate the level of significance of the response relative to the background activity (Wilcoxon rank sum test). The raw traces underlying the data points indicated by the triangles are shown in **c** (gray: individual responses; red: average of 12 responses; black bars above traces: timing of flash). **d** Response reliability of ERG responses (Methods; Supplementary Fig. 1a) to the flashes of 0.79 Michelson contrast (mean ± s.e.m. of *n* = 4 retinas) calculated from the *p*-values in **b**. Even though ERG responses are small at high light levels, they can be reliably detected. Response reliability for flashes of all contrasts is shown in Supplementary Fig. 2

To characterize rod activity in response to different contrast stimuli over a large irradiance range, we first used ex vivo retinas (isolated from RPE) from *Cnga3* ^*–/–*^ mice lacking cone photoreception^16^. With electroretinography (ex vivo ERG, Fig. 1), we recorded the isolated rod response by applying pharmacological agents to inhibit second-order responses in the retina (Methods). During each experiment, we repeatedly presented a set of 50 ms light flashes of four different contrasts (Fig. 1a), while increasing irradiance at 10-fold increments every 30 min. This yielded seven light levels spanning a range from 1 R* rod^–1^ s^–1^ (2 × 10^8^ rod-effective photons cm^–2^ s^–1^) to 10^6^ R* rod^–1^ s^–1^ (2 × 10^14^ rod-effective photons cm^–2^ s^–1^, Fig. 1a). As a measure of response, we compared signal amplitude (mean voltage during 300 ms following flash onset) to baseline (mean voltage during 300 ms before the flash).

**Fig. 2:**
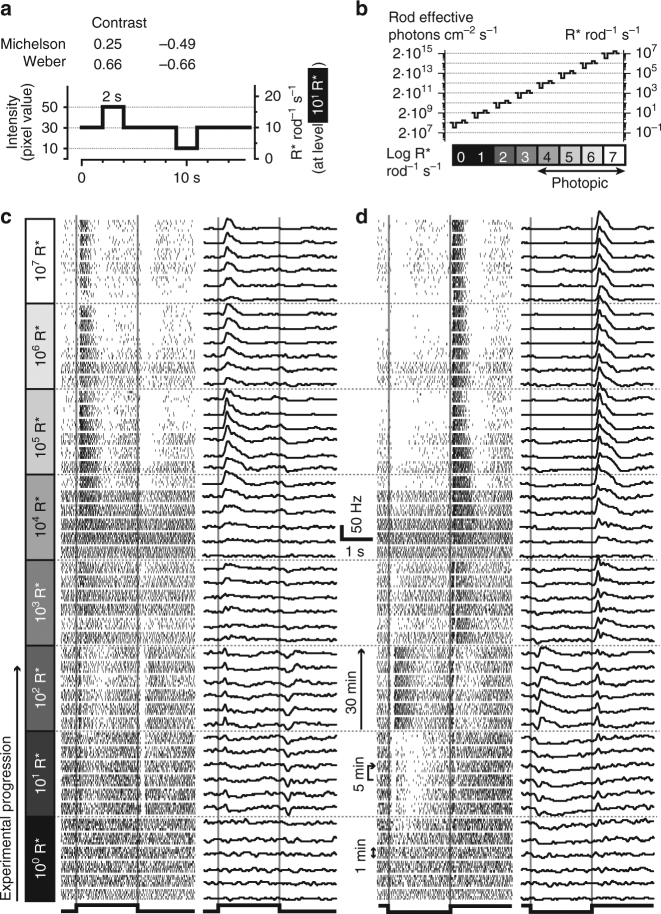
Example responses of a single *Cnga3* ^*−/−*^ ganglion cell across brightness levels. **a** Full-field contrast step stimulus, consisting of positive and negative contrast steps. **b** Absolute intensities of the stimulus shown in **a** at different experimental light levels. **c**, **d** Raster plots (left) and firing rates (right) for a single ganglion cell in response to the full-field positive (**c**) and negative (**d**) contrast steps. Blocks of five consecutive repetitions (left) are averaged in one trace on the right. This cell showed responses at all light levels (very weakly responding at 10^0^ R* rod^−1^ s^−1^), with a short suppression of responses in the beginning of 10^4^ R* rod^−1^ s^−1^. Note that in this ganglion cell, the rod-mediated responses are even stronger at high (10^4^–10^7^ R* rod^−1^ s^−1^) than at lower (1–10^3^ R* rod^−1^ s^−1^) light levels

At lower light levels (≤10^3^ R* rod^–1^ s^–1^), ERGs to all contrasts were detectable (Fig. 1b; Supplementary Fig. 2). At higher light levels (10^4^  to 10^6^ R* rod^–1^ s^–1^), responses to the weaker contrasts (0.33 and 0.6 Michelson) were never detectable, consistent with expected incremental rod saturation^3, 11^. This corresponds to a background at which other authors have described rod saturation^4, 6, 7^ and we therefore refer to 10^4^ R* rod^–1^ s^–1^ and brighter as the “photopic” light range from here on. At the same time, the stimulus with the strongest contrast (0.99 Michelson) always elicited responses at all backgrounds, indicating that rods were never fully saturated in this preparation. During exposure to >10^4^ R* rod^–1^ s^–1^, these responses even gained amplitude over time (Fig. 1b), indicating that rods were becoming more responsive. This latter effect had an absolute impact upon responses to intermediate contrast (0.79 Michelson, red curve in Fig. 1b, individual flash responses shown in Fig. 1c), which were lost upon stepping to photopic backgrounds ≥10^4^ R* rod^–1^ s^–1^, but gradually re-emerged at all irradiances. The time course of response recovery was consistently faster at higher light levels (*n* = 4 retinas, Fig. 1d). Comparable results were obtained from recordings in two other cone-deficient mouse lines (*Pde6c* ^*cpfl1/cpfl1*^ and *Gnat2* ^*cpfl3/cpfl3*^, Supplementary Fig. 2).

The presence of ERG responses to large contrast steps throughout these recordings shows that rods were never fully saturated at any of the backgrounds tested, including at light levels that were well within the photopic range. Moreover, the re-appearance of responses to an intermediate contrast (0.79 Michelson) indicates that the loss of contrast sensitivity was not a fixed property determined by irradiance, but recovered somewhat at all backgrounds. Counter-intuitively, the rate of this recovery was faster at brighter backgrounds.

### Rod-driven ganglion cells responses at all light levels

To understand how such rod activity could impact the retinal output, we turned to multi-electrode array (MEA) recordings of ganglion cell spiking activity from isolated cone-deficient (*Cnga3* ^*–/–*^) retinas (*n* = 10 retinas). Similar to the ERG experiments, we increased background light at 10-fold increments every 30 min, yielding 8 light levels from 1 to 10^7^ R* rod^–1^ s^–1^ (2 × 10^8^ to 2 × 10^15^ rod-effective photons cm^–2^ s^–1^, Fig. 2a, b), and superimposed 2-s full-field steps of positive contrast (+0.25 Michelson, +0.66 Weber) or negative contrast (–0.49 Michelson, –0.66 Weber). The responses of a representative single ganglion cell are shown in Fig. 2c (positive contrast stimulus) and 2d (negative contrast). We presented blocks of five repeats of this stimulus every 5 min (spike raster shown on the left, average spike rate shown on the right). After switching from a background of 10^3^ R* rod^–1^ s^–1^ to a background of 10^4^ R* rod^–1^ s^–1^, the cell shown in Fig. 2c, d did not respond to the stimulus during the first presentation, consistent with the concept that rods would become saturated at higher irradiance. However, just as in the ERG recordings, ganglion cell spiking responses increased over time at this background, such that responses became apparent even to the weak contrast used in this experiment. Furthermore, rather than showing further evidence of saturation, responses of this cell actually became stronger with subsequent increases in irradiance, even up to 10^7^ R* rod^–1^ s^–1^, 1000-fold above the background at which responses had first disappeared with these fixed contrast stimuli.

**Fig. 3:**
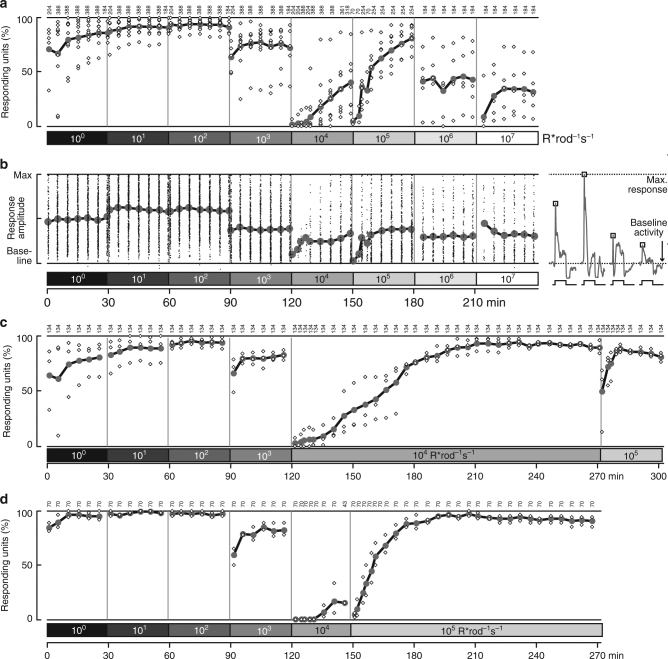
Responsiveness of ganglion cells in isolated *Cnga3* ^*−/−*^ retina. **a** Percentage of responsive ganglion cells in each retina (small white diamonds) and across all experiments (large gray disks and thick line) that responded to a full-field positive or negative contrast step. The numbers on top indicate the total number of ganglion cells recorded at each time point of the experimental paradigm. **b** Response amplitude (normalized peak spike rate) of all individual units that responded (small dots) and their mean response amplitude (large gray dots and thick line). Right panel: Schematic of how response amplitude was determined. Each ganglion cell was followed throughout the experiment, and response strength was taken as the relative peak spike rate (indicated by squares) between the baseline activity of the cell and the cell’s maximal response. For simplicity, schematic shows only the positive contrast step. **c** Percentage of responsive ganglion cells in a subset of experiments (*n* = 3 retinas) in which we stayed at 10^4^ R* rod^−1^ s^−1^ for 2.5 h. Data from these retinas (up to 150 min) are also part of **a** and **b**. **d** Percentage of responsive ganglion cells in a subset of experiments (*n* = 2 retinas) in which we stayed at 10^5^ R* rod^−1^ s^−1^ for 2 h. Data from these retinas (up to 180 min) are also part of **a** and **b**

This behavior was consistent across the population of retinal ganglion cells (Fig. 3). At lower irradiances, most recorded ganglion cells clearly responded to contrast steps (Fig. 3a). Upon initial switch to a background of 10^4^ R* rod^–1^ s^–1^ (2 × 10^12^ rod-effective photons cm^–2^ s^–1^), responses effectively disappeared. However, over time the fraction of responding cells increased again, reaching around 50% after 30 min (Fig. 3a). Similarly, a sizeable fraction of cells retained or recovered responses at even brighter backgrounds (up to 10^7^ R* rod^–1^ s^–1^). Mean response amplitude (across responding cells at each epoch, Fig. 3b) also transiently decreased upon switching to 10^4^ R* rod^–1^ s^–1^ and 10^5^ R* rod^–1^ s^–1^, but otherwise remained at least at 50–70% of the maximal response. Indeed, some ganglion cells, like the one shown in Fig. 2, had their strongest responses at the brightest backgrounds. We observed similar behavior as in Figs. 3a and b in two other cone-deficient mouse lines (*Pde6c* ^*cpfl1/cpfl1*^ and *Gnat2* ^*cpfl3/cpfl3*^, Supplementary Fig. 3).

As seen in Fig. 3a, the fraction of responding cells at 10^4^ R* rod^–1^ s^–1^ and 10^5^ R* rod^–1^ s^–1^ continued to increase throughout the 30 min at these light levels. In a set of experiments, we held retinas at those two backgrounds (10^4^ R* rod^–1^ s^–1^, *n* = 3, Fig. 3c, or 10^5^ R* rod^–1^ s^–1^, *n* = 2, Fig. 3d) for 150 min, and found that nearly all cells eventually regained responses. The major difference between the two light levels was the rate of recovery: it took around 45 min at 10^4^ R* rod^–1^ s^–1^ but only 15 min at 10^5^ R* rod^–1^ s^–1^ for 50% of the ganglion cells to recover responses. Thus, recovery was faster at a brighter photopic background.

These ex vivo ganglion cell recordings from cone-deficient mice revealed that rod responses at photopic light levels can drive ganglion cell spiking activity, even at contrasts that do not elicit reliable ERG signals. In other words, the ability to detect rod-driven responses at high backgrounds to a stimulus with defined contrast may depend on the sensitivity of the recording method in addition to the photoreceptor biology. The time course of ganglion cell responsiveness further confirms that high background intensities abolish responses to moderate contrast stimuli, but those responses recover over time such that rods can drive retinal activity to moderate contrasts across all irradiances tested. Furthermore, once within the “photopic” range, increasing irradiance can actually enhance rod-driven ganglion cell responses in the isolated retina.

### Rod-driven thalamic responses at all light levels in vivo

**Fig. 4:**
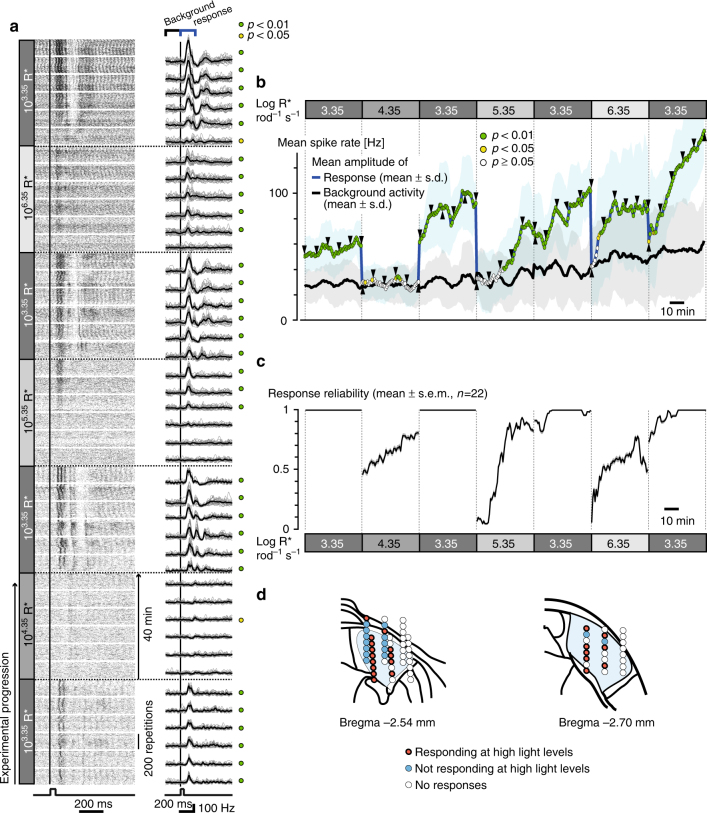
Rod responses in in vivo dLGN recordings in *Cnga3* ^*−/−*^. **a** Example response of one multiunit to a 50 ms flash at different light levels. Responses to each of the 8400 single repetitions are shown in the raster plot (left). For analysis, 10 consecutive repetitions were averaged (one “group”, gray lines on the right). In black, the averages over 20 such groups (200 flashes) are shown. **b** Response amplitude (blue curve) and background activity (black curve) of the multiunit shown in **a** (moving average over runs of 20 groups (=200 flashes), shifted by one group. Arrowheads mark values corresponding to the raw traces shown in the right column in **a**). Responses significantly above background are color-coded in green (*p* < 0.01) and yellow (*p* < 0.05, rank sum test). This example multiunit stops responding after switching to 10^4.35^ R* rod^−1^ s^−1^. However, at 10^5.35^ and 10^6.35^ R* rod^−1^ s^−1^ the responses reappear after several minutes. **c** Population data for those multiunits responding at high light levels (*n* = 22/36, mean ± s.e.m.), depicting the reliability of their responses (Methods; Supplementary Fig. 1a). Responses at high light levels recovered with an intensity-dependent time course. **d** Electrode positions during LGN recordings. Electrodes on which responses recovered at high light levels are color-coded in red, electrodes with responses only at moderate light levels are colored in blue

Having recorded rod-driven responses in explanted retinas under even the brightest backgrounds, we next asked whether rods may mediate such light responses also in vivo. To this end, we recorded multiunit activity from the dorsal lateral geniculate nucleus (dLGN) of anesthetized *Cnga3* ^*–/–*^ mice (Fig. 4, recording positions shown in Fig. 4d) in response to 50 ms flashes of positive contrast (0.75 Michelson). In these experiments, higher (photopic) irradiances (10^4.35^, 10^5.35^, and 10^6.35^ R* rod^–1^ s^–1^) were interspersed with moderate irradiance (mesopic, 10^3.35^ R* rod^–1^ s^–1^, 4.52 × 10^11^ rod-effective photons cm^–2^ s^–1^, predicted to support strong rod responses), to confirm that the mice retained good visual responses throughout the recording session.

Firing patterns of a representative multiunit recording are shown in Fig. 4a. As predicted, strong and stable flash responses were recorded at the mesopic background of 10^3.35^ R* rod^–1^ s^–1^ (4.52 × 10^11^ rod-effective photons cm^–2^ s^–1^). A 10-fold increase of irradiance (to 10^4.35^ R* rod^–1^ s^–1^, 4.52 × 10^12^ rod-effective photons cm^–2^ s^–1^) made responses hard to discern. Upon further increases of irradiance, responses were suppressed at first, but re-emerged over time. Similar to the ex vivo recordings, the rate of response recovery was positively correlated with irradiance. These general patterns were confirmed by a systematic analysis of these data (Fig. 4b), in which response amplitude (difference in spike rate in 200 ms windows before and after the flash) and reliability (based on *p*-value for rank sum test comparing these values) were plotted as a function of time.

Most recorded multiunits (*n* = 22/36) exhibited a similar pattern of transient response loss and recovery at the two highest backgrounds, 10^5.35^ and 10^6.35^ R* rod^–1^ s^–1^, with the time course of recovery being faster at 10^6.35^ R* rod^–1^ s^–1^ (Fig. 4c). Other multiunits (*n* = 14/36) failed to respond consistently at those irradiances (*p* > 0.05 for rank sum test for at least half of the trials). Behavior at the intermediate irradiance (10^4.35^ R* rod^–1^ s^–1^) was variable, with some multiunits matching the very poor responses shown for the single example in Fig. 4a, b, while others responded reliably (Fig. 4c).

These results in LGN of cone-deficient mice (Fig. 4) were consistent with ganglion cell responses in isolated retina (Fig. 3): Responses transiently disappeared when switching to higher photopic light levels, and re-emerged with a time course which was faster for higher background intensities. Our ex vivo ERG recordings (Fig. 1) suggested that this transient lack of responsiveness may be due to incremental saturation, i.e., reduced contrast sensitivity, rather than absolute saturation. We next tested if this interpretation would also apply to the in vivo situation.

**Fig. 5:**
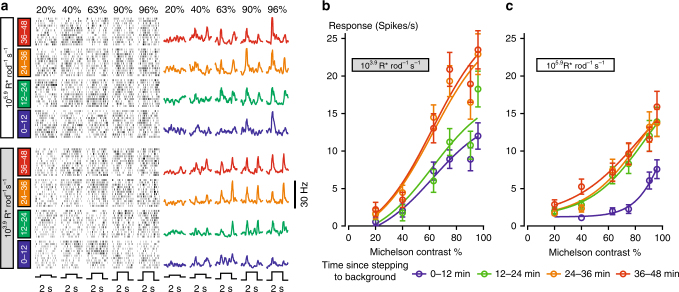
Incremental saturation in rod-driven responses in the *Cnga3* ^*−/−*^ LGN. **a** Example peri-event rasters (left) and peri-event histograms (right) for a single LGN multiunit in response to spectrally neutral modulations in background light intensity across a range of contrasts (different columns) and at various time points (different colors, range in min given on the left) after stepping to that background. **b**, **c** Mean ± s.e.m. contrast–response relationship across 86 LGN multiunits (from four mice) at varying time points after stepping to the 10^3.9^ R* rod^−1^ s^−1^ background (**b**, from darkness) or 10^5.9^ R* rod^−1^ s^−1^ (**c**, from 10^3.9^ R* rod^−1^ s^−1^). Continuous curves represent the best-fitting 4-parameters sigmoid curves

After stepping to a low photopic background intensity (10^3.9^ R* rod^–1^ s^–1^), we presented square-wave contrast steps (0.25 Hz) at six different contrasts, centered around the mean background intensity (Fig. 5). While presenting these contrast steps for a total of 48 min, we analyzed the peak-to-trough responses of 86 recorded LGN multiunits in four time bins of 12 min each. During the first 12 min after stepping to this background intensity (Fig. 5b, shown in blue), the weakest contrast stimuli (20% Michelson) did not elicit responses, while stronger-contrast stimuli did. Over time, responses for all stimuli grew in amplitude, reaching steady state during the third time bin (i.e., after about 30 min, shown in orange). Also the 20%-contrast stimulus elicited noticeable responses at that time.

We then stepped to a higher photopic background (10^5.9^ R* rod^–1^ s^–1^, Fig. 5c), repeating the same stimulation paradigm. At this background, the reduction in contrast sensitivity during the first 12 min was more pronounced, with stimulus contrasts of 70% or higher being necessary to elicit responses. However, responses recovered also at this background, reaching steady state already during the second time bin (shown in green), i.e., faster than at 10^3.9^ R* rod^–1^ s^–1^.

In summary, LGN multiunits exhibited response features upon stepping to photopic background levels which were consistent with those of ERG and ganglion cell recordings ex vivo, namely initially reduced contrast sensitivity with faster recovery at brighter light levels.

### Rods shape photopic thalamic responses in the presence of cones

**Fig. 6:**
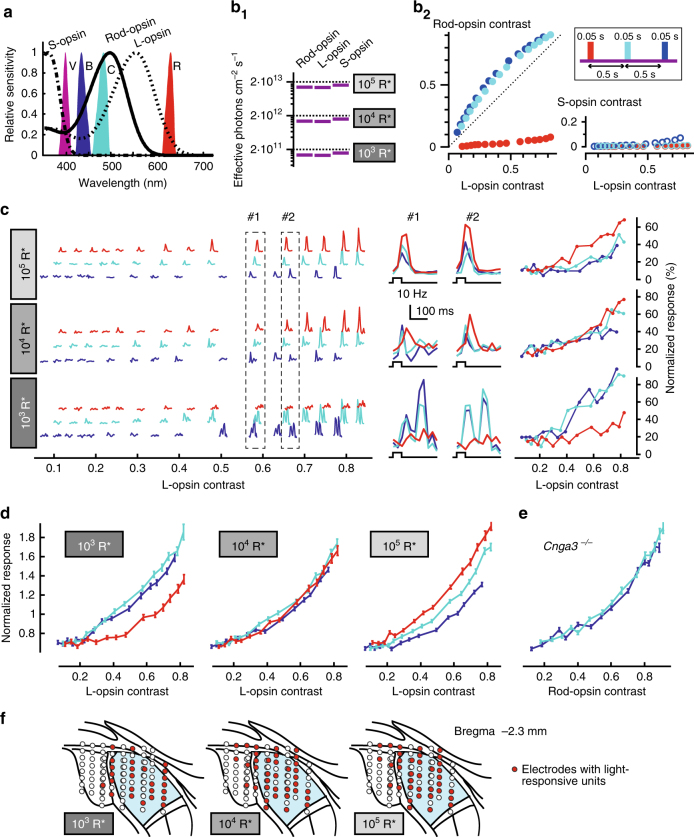
Photopic LGN activity in vivo is influenced by rods in the presence of cones. **a** Spectral power of stimuli and photoreceptor spectral sensitivity in *Opnmw* ^*R*^ *;Opn4* ^*−/−*^ mice. S-opsin, rod-opsin, and L-opsin are roughly equally sensitive to the violet (V) background, but have markedly different sensitivity to blue (B), cyan (C), and red (R) flash stimuli. **b** Stimulus intensity and contrast. **b**_**1**_ Estimated absolute intensity of the violet background (retinal irradiance). Dashed lines correspond to 10^3^, 10^4^, and 10^5^ R* rod^−1^ s^−1^. **b**_**2**_ Michelson contrast of blue, cyan, and red flash stimuli. They present similar contrast for L-opsin, while rods are hardly activated by red stimuli. All stimuli present very weak contrast to S-opsin. Inset: timing of stimulus flashes. **c** Responses of an example unit at different irradiances. Left: mean firing rate for stimuli of all presented contrasts. Middle: expanded comparison of responses to blue, cyan, and red stimuli of roughly equivalent L-opsin contrast. Right: normalized mean response amplitude (Methods) as function of L-opsin contrast. **d** Mean ± s.e.m. of response amplitude, normalized by the mean response across contrasts and colors (Methods), to blue, cyan, and red flashes as a function of L-opsin contrast for all light-responsive units at different irradiances. Responses at the three wavelengths could be fit with a single function at 10^4^ R* rod^−1^ s^−1^ (consistent with the view that they are driven by L-opsin), but not at lower or higher irradiances (see Supplementary Table 1 for statistical analysis of curve fits). **e** Mean ± s.e.m. of normalized response amplitude (Methods) for cyan and blue flashes presented to *Cnga3* ^*−/−*^ mice at 10^2^ R* rod^−1^ s^−1^ as a function of estimated rod contrast (*n* = 229 responsive units from three mice; two different electrode placements in mice 1 and 2, three placements in mouse 3). The responses at the two colors were indistinguishable confirming the suitability of our methods for estimating photoreceptor spectral sensitivity in vivo (BC: *R* ^2^_BC_ = 0.448, *R* ^2^_BC,null_ = 0.448, ∆*R* ^2^_BC_ ≈ 0). **f** Histological confirmation for electrode placements in *n* = 3 *Opnmw* ^*R*^ *:Opn4* ^*−/−*^ animals

The experiments with cone-deficient mice revealed that rods can function across all physiological background light intensities. The difficulty of observing their activity with ERG (Fig. 1) suggests that their light responses are weak, even though they apparently can activate downstream pathways reliably (Figs. 2–5). A reasonable question is whether this allows rods to contribute to visual responses in animals with an intact visual system (i.e., when cones are also functional), or whether the gain of rod signals compared to cone signals is so small that the rod contribution is negligible. To answer this, we set out to measure rod contribution to the overall visual response in mice with intact cone function by using a transgenic mouse line (*Opn1mw* ^*R*^) in which the mouse M-opsin coding sequence is replaced by the human long-wavelength sensitive (“L” or “Red”) opsin sequence^17^. In this animal, the wavelength sensitivities of rods and the two cone types are very different (Fig. 6a), allowing us to test if rods alter the spectral sensitivity of visual responses under bright backgrounds. To rule out the possibility of recording melanopsin-driven responses, we crossed these *Opn1mw* ^*R*^ animals with a melanopsin knockout (*Opn4* ^*–/–*^) line^18^ (*Opn1mw* ^*R*^ *:Opn4* ^*–/–*^ mice).

Anesthetized *Opn1mw* ^*R*^ *:Opn4* ^*–/–*^ animals were adapted to a violet light (*λ*_max_ = 400 nm) to which rods and cones containing L-opsin and S-opsin are approximately equally sensitive (Fig. 6a, b_1_). Responses to blue (*λ*_max_ = 430 nm), cyan (*λ*_max_ = 480 nm), and red (*λ*_max_ = 630 nm) flashes presented in pseudorandom order at 15 different intensities superimposed upon the violet background (Fig. 6b_2_) were recorded in the contralateral dLGN (recording positions shown in Fig. 6f). Due to the divergence in spectral sensitivity between rhodopsin, S-opsin, and L-opsin, there is a big difference in the effective contrast of these flashes for rods and cones. In particular, while flashes at all three wavelengths present significant contrast for L-opsin, rods should be much less responsive to red stimuli, and S-opsin contrast is very weak at all wavelengths (Fig. 6b_2_). Under the assumption that rods are in absolute saturation and only cones are responding, we would expect equivalent responses to flashes at all wavelengths when flash strength is expressed in units of L-opsin contrast. On the other hand, rod intrusion at mesopic irradiances should produce differential responses to blue and cyan vs. red flashes. For example, a blue and a red flash with equal L-opsin contrast should nevertheless produce different responses at mesopic light levels, because to rods, the blue flash appears brighter than the red flash.

We found clear evidence of rod intrusion in the composite flash responses when these flashes were presented on a violet background generating ~10^3^ R* rod^–1^ s^–1^ (1.38 × 10^11^ rod-effective photons cm^–2^ s^–1^), at which rod responses were always strong in our recordings with cone-deficient mice (Fig. 4). Specifically, when flash intensities were expressed in units of L-opsin contrast, responses were consistently larger for blue and cyan flashes than for red flashes, as shown in Fig. 6c for a single unit and in Fig. 6d for the population of recorded units (see Supplementary Table 1a for statistical analysis). Upon stepping up to 10^4^ R* rod^–1^ s^–1^, this difference disappeared, with response amplitude to flashes at all wavelengths being adequately predicted by their L-opsin contrasts. Responses at 10^4^ R* rod^–1^ s^–1^ could thus be interpreted as being solely cone-driven. Consistent with the other data presented here, however, a further increase in irradiance (to 10^5^ R* rod^–1^ s^–1^) produced an increase in rod intrusion. At 10^5^ R* rod^–1^ s^–1^, flash response amplitude could no longer be predicted by L-opsin contrast. In this case, blue and cyan responses were consistently smaller than red responses of similar L-opsin contrast. These data indicate an inhibitory influence of rods on the cone flash response at this high photopic level. Such inhibitory rod–cone interactions have precedent in the psychophysics literature^19^.

To confirm that the effect at 10^5^ R* rod^–1^ s^–1^ was not attributable to some methodological error, we first tested how robust it was to errors in our estimate of in vivo L-opsin spectral sensitivity. We varied the two parameters that could strongly influence this estimate (pigment optical density and pre-receptoral spectral filtering; Methods), but found that the reduced responsiveness at blue and cyan was retained (Supplementary Table 1b). We next tested if responses to blue and cyan flashes in cone-deficient *Cnga3* ^*–/–*^ mice were equivalent to each other when expressed in units of our estimated rod-opsin contrast, which was the case (Fig. 6e). This suggests that the difference to blue and cyan flashes observed at 10^5^ R* rod^–1^ s^–1^ in *Opn1mw* ^*R*^ *:Opn4* ^*–/–*^ mice (Fig. 6d) could be attributable to a difference in relative excitation of rods and L-opsin (L-opsin to rod-opsin ratio of excitation at brightest flash: 0.48 for blue and 0.44 for cyan). In summary, while our data are consistent with flash responses being wholly cone generated at 10^4^ R* rod^–1^ s^–1^, they reveal a significant rod contribution at the brighter background (10^5^ R* rod^–1^ s^–1^).

### Comparison with computational model of rod phototransduction

**Fig. 7:**
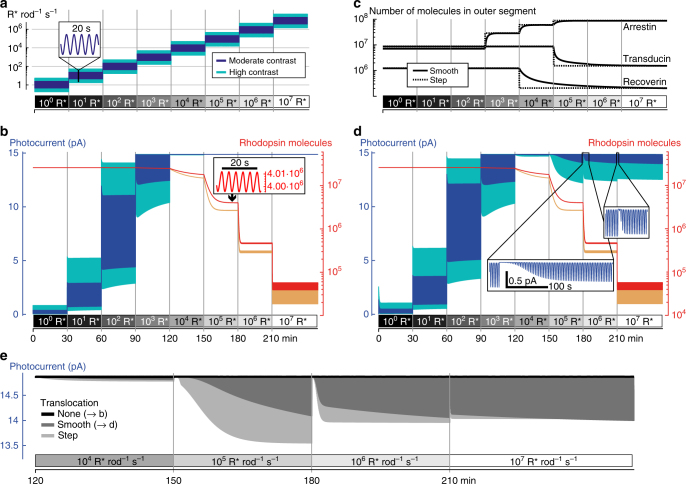
Computational model of rod light responses. **a** Stimulus used for modeling was a sinusoid (0.25 Hz) of either moderate (blue) or high (cyan) amplitude (full amplitude 0.75 or 1.5 log units; Michelson contrast 0.7 or 0.94). Every 30 min, the ambient level increased by 1 log unit, ranging from 1 R* rod^−1^ s^−1^ to 10^7^ R* rod^−1^ s^−1^. **b** Behavior of the original model by Invergo et al. with few adjustments (Methods). Left *y*-axis: elicited photocurrents to moderate (blue) or high (cyan) contrast stimulus; right *y*-axis, logarithmic: number of unbleached rhodopsin molecules in outer segment to moderate (red) or high (orange) contrast stimulus. Inset: Detailed view of number of rhodopsin molecules. **c** Implementation of arrestin, transducing, and recoverin translocation as smooth kinetic processes (solid lines) or as step-like events (dashed lines). **d** Model behavior as in **b**, with translocation of arrestin, transducing, and recoverin modeled as smooth kinetic processes. Both insets are plotted on the same scale. **e** Photocurrents at photopic light levels to the moderate contrast stimulus with different implementation of transducin, arrestin, and recoverin translocation. Black: no translocation (replicated blue curve from **b**). Dark gray: smooth translocation (replicated blue curve from **d**). Light gray: step-like translocation

What physiological processes could explain the observed pattern of rod responses under bright backgrounds? We probed the most detailed available computational model of rod phototransduction^20^ (simulating phototransduction with a system of ordinary differential equations (ODE)) with a sinusoidal stimulus (0.25 Hz) of either strong contrast (0.94 Michelson, full amplitude 1.5 log units) or moderate contrast (0.7 Michelson, full amplitude 0.75 log units, Fig. 7a) while increasing the background every 30 min by 1 log unit, spanning low to high intensities (1 to 10^7^ R* rod^–1^ s^–1^). The model predicted saturation (lack of photocurrent modulations) at the higher irradiances used in our experiments (Fig. 7b; blue and cyan curves show the predicted photocurrents to the moderate or strong contrast stimulus, respectively). However, while the model predicted rod saturation at high light levels, modulations in the concentration of unbleached rhodopsin (red and orange curves for moderate and strong contrast) were retained at all irradiances (see inset in Fig. 7b). This indicates that if the model incorporated a reduced gain of the phototransduction cascade, this might prevent saturation and allow rods to respond to this stimulus.

The original model’s parameters were fitted to physiological recordings from mouse rods under dark-adapted conditions. They thus did not account for any aspect of photoreceptor light adaption, such as well-established irradiance-dependent translocation of elements of the phototransduction cascade between inner and outer segments^21, 22^. For example, at higher light levels, arrestin moves into the outer segment, increasing its effective concentration; while transducin and recoverin leave the outer segment, reducing their concentration. These translocations have the net effect of reducing the gain of the phototransduction cascade, thereby contributing a mechanism of light adaptation. We modified the model to include these translocation events (Fig. 7c, solid lines). This modification resulted in modulated photocurrents at all light levels (Fig. 7d).

Including translocation of phototransduction elements allowed the model to replicate our three main experimental observations: First, responses at photopic light levels (10^4^ R* rod^–1^ s^–1^ and above) are initially weak when stepping to that background, but recover over time. Second, saturation was less apparent for stronger stimulus contrast (compare cyan and blue traces in Fig. 7d). Third, the time course of response recovery was faster at higher light levels (see also magnified views in Fig. 7d). Thus, translocation of transducin, arrestin, and recoverin in the model was crucial to enable photocurrents at high light levels. However, the specific time course of the translocation events was less important. We tested the effects of implementing the translocations as artificial, step-like events (dashed lines in Fig. 7c) instead of the more realistic kinetic processes (solid lines in Fig. 7c). As expected, such immediate concentration changes resulted in more rapid recovery in photoresponse (light gray vs. dark gray curves in Fig. 7e), but this did not change the qualitative observation that responses recovered faster at higher photopic light levels. In other words, the light-level-dependent time course of photocurrent recovery needs to be influenced by an additional component, beyond the translocation events. Interestingly, faster response re-emergence at higher irradiances coincided with the increased rate of rhodopsin bleaching (red curve in Fig. 7d). Rhodopsin bleaching reduces the rate of isomerization events and might thus be one of the mechanisms allowing for rod responses at high light levels, similar to the suggested role of bleaching adaptation in cones^23^.

### Bleaching adaptation supports photopic rod responses

**Fig. 8:**
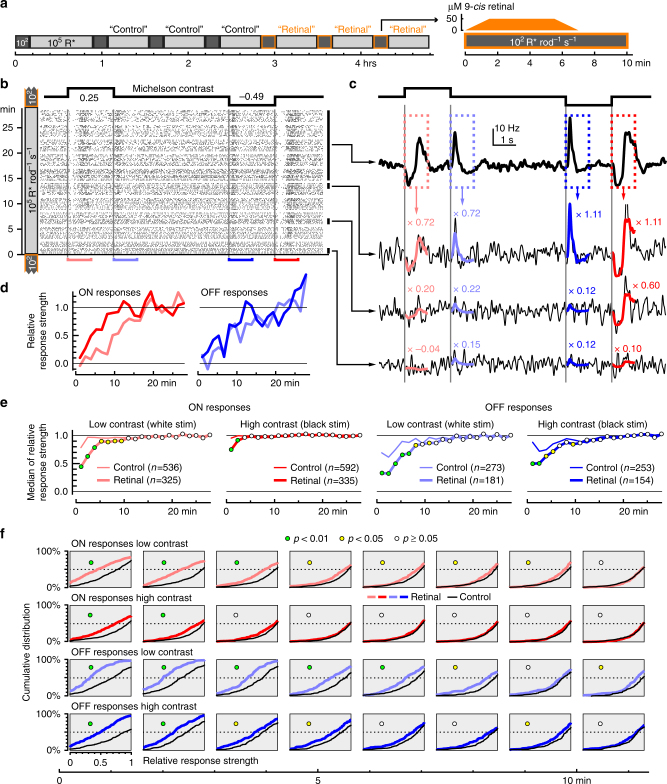
Bleaching adaptation supports recovery of contrast sensitivity at high irradiance. **a** Experimental protocol. Ganglion cell responses in *Cnga3* ^*−/−*^ retinas were tested at high light levels (10^5^ R* rod^−1^ s^−1^), with brief (10 min) interruptions with dim light (10^2^ R* rod^−1^ s^−1^). During the last three dim segments, 50 µM 9-*cis* retinal was applied. **b**–**d** Relative response strength of one example ganglion cell to 2-s positive and negative contrast steps (same stimulus as in Fig. 2b), during one experimental segment at 10^5^ R* rod^−1^ s^−1^ after 9-*cis* retinal application. **b** Spike raster. **c** Average firing rate to the last 50 repetitions of the stimulus (top) was used as a template to quantify the evolution of response strength in this experimental segment by linearly fitting the first 1000 ms of the cell’s response (average firing rate to five stimulus repetitions) after each contrast transition (ON stimuli of low and high contrast, light red and red; OFF stimuli of low and high contrast, light blue and blue). **d** Evolution of response strength of this cell during this experimental segment, for all four contrast transitions. **e**, **f** Median relative response strength (**e**) and cumulative distribution of relative response strength (**f**, for the first eight time points shown in **e**) across all experimental segments and all ganglion cells from four retinas recorded with an experimental protocol as in **a**, and two retinas recorded with a similar protocol without supplying 9-*cis* retinal. Test for statistical significance was performed with a permutation test (Methods). Note that the relative response strength can be <0 and >1 (see examples in **c**, **d**), so that the cumulative distribution does not fully cover the range from 0 to 100% in the panels shown in **f**

To directly test if rhodopsin bleaching might indeed support rod responses at higher light levels, we returned to ex vivo MEA ganglion cell recordings from retinas of cone-deficient *Cnga3* ^*–/–*^ mice. We explored the impact on bright light responses of supplying exogenous chromophore (50 µM 9-*cis* retinal, facilitating rhodopsin regeneration). A technical challenge in achieving this was that the bright background light would itself bleach exogenous chromophore. We therefore devised a protocol in which ganglion cell responses were recorded at a high light level (10^5^ R* rod^–1^ s^–1^), but with occasional interruptions by 10 min at low mesopic levels (10^2^ R* rod^–1^ s^–1^) during which chromophore could be applied (Fig. 8a). While application of chromophore is generally considered to support visual responses in the ex vivo preparation, one would expect the opposite in the case of bleaching adaptation, with responses to contrast steps at the bright background suppressed following epochs of chromophore application.

In accordance with our earlier results (Fig. 3), ganglion cell responses disappeared when first switching to 10^5^ R* rod^–1^ s^–1^ in this paradigm, and recovered within about 15–30 min. Then, during the intermittent 10 min periods at dim background (10^2^ R* rod^–1^ s^–1^), ganglion cell responses were completely absent, consistent with loss of sensitivity of bleached rods to dim stimuli. Upon stepping back to 10^5^ R* rod^–1^ s^–1^ from the dim background, however, contrast responses of ganglion cells were suppressed only weakly and briefly (Fig. 8e, f, control). Conversely, when adding 9-*cis* retinal during the dim period, responses of some ganglion cells recovered toward the end of the dim period, indicating that the lack of responses in the corresponding control trials was at least partly attributable to rhodopsin bleach. After returning to 10^5^ R* rod^–1^ s^–1^, however, responses were significantly weaker than in control conditions (Fig. 8e, f, retinal). This indicates that enhanced regeneration of rhodopsin had detrimental effects on ganglion cell responses at high light levels, consistent with the hypothesis that rhodopsin bleaching is indeed one of the mechanisms that allow rods to escape saturation in bright backgrounds. Interestingly, responses to lower contrast stimuli were more strongly affected by rhodopsin regeneration than responses to higher contrast (Fig. 8e, f), reinforcing our earlier observations (Figs. 1, 5) that saturation is expressed as reduced contrast sensitivity.

## Discussion

Here, we characterized rod-driven visual responses in the mouse over a large irradiance range, from scotopic to high photopic light levels. All our data—ERG and ganglion cell recordings from isolated retina ex vivo, and recordings of LGN responses in vivo—shows that rods can drive visual responses to moderate contrast stimuli, within the range encountered regularly during natural viewing^24^, at all physiologically relevant irradiances. It further shows that, at photopic backgrounds, this capacity is associated with a recovery in contrast sensitivity over extended exposure, and that this recovery occurs faster at higher backgrounds. Such rod-driven responses were not only readily observed in cone-deficient mice, but also contributed to the visual responses in visually intact animals. Together, our data show that the presence and re-appearance of rod responses depends not simply on the background intensity, but on the interaction between background intensity and the duration of exposure. Surprisingly and counter-intuitively, both higher background intensity and prolonged exposure result in reduced saturation, i.e., in more robust rod-driven signaling.

We initially aimed to use transgenic cone-deficient mice to describe the transition to rod saturation. The concept of rod saturation at bright backgrounds is widely accepted^25–29^, and dates back at least to the classical study of Aguilar and Stiles^3^, who found incremental rod saturation with the psychophysical two-color incremental threshold test. Green^4^ measured ERG responses from rat retina and found rod saturation with a two-color incremental threshold test similar to the one used by Aguilar and Stiles. However, an important aspect of both studies, often overlooked, is that they report loss of contrast sensitivity rather than full rod saturation. Our own work highlights the importance of this distinction. We too see loss of rod contrast sensitivity when stepping to bright backgrounds, but this does not correspond to full rod saturation. Rather, rod responses were always apparent if stimuli of sufficient contrast were applied.

When interpreting the results of visual experiments carried out at high backgrounds, especially when considering whether rods contribute to visual processing, several factors should be considered. First, in addition to stimulus contrast, is the sensitivity of the recording method: stimulus-evoked rod responses (Fig. 3) may not be apparent with a less sensitive method, such as ERG (Fig. 1)^30, 31^. Second, thanks to the recovery of rod contrast sensitivity over time at photopic backgrounds, rod responses to moderate contrasts may re-emerge even after lacking for many minutes. Third, even more importantly, the recovery rate in contrast sensitivity is itself positively correlated with irradiance. High backgrounds are commonly used as an experimental strategy to study cone vision. Our experiments confirm that stepping to high backgrounds can indeed produce retinal responses that are strongly or even exclusively cone-driven. However, they further reveal that the duration of this exclusively cone-driven operational regime is shortest at higher light levels. Thus, care needs to be taken to limit exposure to photopic light levels if the aim is to exclude rod responses^32^. Indeed, it is not safe to assume that rods stay silent at any background light intensity, and further irradiance increase, once in the photopic range, can actually be detrimental to the objective of isolating cone vision. Here, we were able to observe rod intrusion at high light levels in visually intact animals due to the distinct spectral sensitivities of rhodopsin and the cone opsins in the “red opsin” mouse model. Since wild-type mice have very similar spectral sensitivity of rhodopsin and M-opsin, unwanted rod intrusion at high light levels can easily go unnoticed.

The results of the computational modeling are instructive with respect to the potential mechanisms that allow rods to signal at even the brightest light levels. A previously published model of rod phototransduction^20^ predicted that rhodopsin isomerization was triggered by visual stimuli at all backgrounds (red curve, Fig. 7b), but that this was not translated into changes in membrane current at irradiances causing 10^4^ R* rod^–1^ s^–1^ or more (blue curve, Fig. 7b). Inclusion of irradiance-dependent translocation of transduction cascade components between inner and outer segment (transducin, arrestin, and recoverin, Fig. 7c) aligned the model with our physiological data (Fig. 7d). The net effect of the translocations is a reduced gain of the cascade, such that the stimulus-induced variation of activated rhodopsin was translated into a modulation of photocurrent. According to the model, then, these translocations are a prerequisite for rod responses at high backgrounds. They do not explain all aspects of such activity, however, as even when we modeled these translocations as instantaneous events, the model recreated the slow build-up of rod responses under extended exposure to high irradiance and the positive correlation between irradiance and the rate of this recovery (Fig. 7e). In the model, the response recovery rate coincided with the rate of rhodopsin bleaching, which naturally occurs more rapidly at higher irradiance. Since rhodopsin bleaching also results in a reduced gain of the transduction cascade, it might contribute a form of “bleaching adaptation” in rods equivalent to that previously described for cone photoreceptors^23^. Our experiments confirmed this hypothesis (Fig. 8). Note, however, that bleaching can also have the opposite effect and contribute to saturation, because bleached rhodopsin (i.e., opsin) slightly activates the phototransduction cascade^33, 34^.

The description of bleaching adaptation raises an interesting further question. Why did our ex vivo preparation not stop responding to light simply because all its visual pigment had become bleached? We were able to record responses from cone-deficient retinas isolated from the retinal pigment epithelium (RPE) for many hours at high light levels that should have fully bleached available rhodopsin in minutes or even seconds of exposure. While cones can take advantage of 11-*cis* retinol generated in Müller cells, which they convert to 11-*cis* retinal^31, 35, 36^, rods are thought to depend on the RPE visual cycle. How can rods then retain responsiveness? Kaylor et al.^37^ recently showed that all-*trans* retinal, after having dissociated from opsin, can regenerate to 11-*cis* retinal within the membrane phospholipid bilayer of photoreceptors in a light-driven reaction. This process would naturally be strongest at high light levels, resulting in the maintenance of a steady-state pool of regenerated rhodopsin. Alternatively, our ex vivo retina preparation may retain sufficient RPE (we occasionally see small specks of pigment epithelium remaining on the isolated retina) to recycle chromophore.

The mathematical model allows exploration of rod behavior at high irradiance with variations in chromophore recycling. Higher rates of chromophore recycling (model parameter *k*_Rrecyc_) abolish responses at high irradiance (Supplementary Fig. 4, first row), consistent with the proposed importance of bleaching adaptation, and consistent with results of our chromophore augmentation experiments (Fig. 8). Interestingly, strong reductions in *k*_Rrecyc_ (below 1% of the standard parameter value used in Fig. 7a–e) had the same effect, owing to excessive accumulation of opsin, which saturated the cascade^33, 34^. In essence, only intermediate values of *k*_Rrecyc_ predicted photopic rod responses (Supplementary Fig. 4, blue curves in panels 2–4). Could changes in other key model parameters support light responses at high backgrounds, despite a lower chromophore regeneration rate likely encountered in ex vivo experimental conditions? The behavior at high irradiance was also dependent upon both the total number of rhodopsin molecules (parameter *Rhod*_total_, columns in Supplementary Fig. 4) and the efficiency with which opsin (bleached rhodopsin) activates the cascade (parameter *k*_Ops_, different colored curves in Supplementary Fig. 4). Lower values for either parameter supported photopic responses, allowing the model to match responses recorded ex vivo at lower values for *k*_Rrecyc_.

Therefore, the sensitivity of rod responses at high irradiance could depend on naturally occurring variations in the properties of the phototransduction cascade and its regulation (e.g., differences in molecular concentrations, kinetic properties, translocation processes, or outer segment volume). Such variations might exist between species, between individuals of the same species, between different rods in the same retina, or even within the same rod across the circadian cycle. This observation might further explain discrepancies in the literature concerning the rod saturation threshold (Introduction).

The updated computational model presented here provides a hypothetical biochemical explanation of our experimental observations. All of our adjustments to the original Invergo et al.^20^ model were based on reported biophysical rod properties and light adaptation events in rods. Thus, we did not fit the model to our data, but found that implementing already well-established adaptational processes resulted in a surprisingly good qualitative match between the model behavior and our experimental observations. Explaining rod responses at high light intensities does therefore not require any additional mechanisms beyond what is already known about rod behavior. Nevertheless, additional consequences of bright light exposure may well exist. It was recently shown^38^ that reduced sensitivity of the phototransduction cascade after strong bleaching is partly compensated by properties of the rod inner segment to achieve more robust voltage responses. Furthermore, the supramolecular organization of rhodopsin and transducin into clusters on the disc membrane has been proposed to be important for fast and reliable light responses^39–41^. In the context of bright backgrounds, with reduced concentration of both rhodopsin (due to bleaching) and transducin (due to translocation), regulation of such scaffolding might contribute to controlling the gain of photoresponses.

Do these conclusions drawn for mice translate to other species, including humans? The most direct proof for rod-mediated vision under photopic conditions would be obtained by psychophysical tests with rod monochromats. This is difficult due to the common photophobia of such individuals when exposed to bright light^42^. However, several studies showed that rods impact aspects of color perception at scotopic and mesopic light levels in human trichromats^43–45^, dichromats^46^, and blue cone monochromats^47^ (reviewed by Zele and Cao^48^). This influence of rod signaling on color vision could be used to test for rod contribution to visual perception also under bright light conditions. Further, insights into rod-mediated vision under bright light can also be gained form a subset of rod-monochromatic individuals, which are not blinded in such an environment^49^. It is interesting that the rod system in these individuals is apparently less prone to saturation. According to our model, this could be explained by differences in rod biochemistry in these particular patients resulting in different gain control. Deeper insight into the underlying mechanisms of this effectively non-saturating phenotype might reveal new opportunities to treat rod monochromats by appropriately interfering with the properties of the rod cascade, potentially by reducing the gain. While such a reduced gain would be counterproductive for low-light vision, our daily lives, with electrical lighting all around us, happen mostly beyond the scotopic range, so that such treatment could indeed have a net positive benefit.

## Methods

### Animals

We used several transgenic mouse lines in which cone responses are abolished due to mutations disrupting the cone phototransduction cascade. In *Cnga3* ^*–/–*^ mice^16^, kindly provided by M. Biel for ex vivo experiments, the cone-specific alpha-subunit of the cyclic nucleotide gated channel is mutated, preventing voltage changes in cones upon light activation. *Cnga3* ^*–/–*^ mice were 4.5–6 weeks old for ganglion cell recordings, 8 weeks for ex vivo ERG recordings, and ~6–8 weeks for in vivo experiments. In *Pde6c* ^*cpfl1/cpfl1*^ mice (Jackson strain #3678), kindly provided by Bo Chang (The Jackson Laboratory, Bar Harbor, ME), the cone-specific phosphodiesterase is non-functional. *Pde6c* ^*cpfl1/cpfl1*^ mice were 11–13 weeks old. In *Gnat2* ^*cpfl3/cpfl3*^ mice (Jackson strain #6795), the cone-specific transducin is non-functional. *Gnat2* ^*cpfl3/cpfl3*^ mice were 5–13 months old, only data from animals at least 1 year old are shown here. All ex vivo experiments were performed with explanted retinas, with RPE removed. A characteristic feature in these preparations was the transient suppression of responses to moderate contrast stimuli upon switching to photopic light levels (10^4^ R* rod^–1^ s^–1^ and brighter), a feature not observed in wild-type retinas with functional cones. Further, the *Gnat2* ^*cpfl3/cpfl3*^ model has slow cone degeneration with cones surviving up to 1 year. In contrast to older animals (12–13 months), younger animals (5 months) did not have complete response suppression after switching to photopic light levels, which indicates residual cone function. In other words, the complete response suppression to moderate contrast stimuli in experiments described here suggests that cones were indeed non-functional in those retinas and responses, when present, were rod-driven. “Red opsin” mice (Opn1mwR; Opn4^–/–^) of ~6–18 weeks were used for in vivo experiments and bred in-house at the University of Manchester, UK.

Animal use was in accordance with German, UK, and European regulations and approved by the Regierungspräsidium Tübingen (ex vivo experiments) and the local Manchester Animal Welfare and Ethical Review Board (AWERB; Manchester, UK; in vivo experiments).

### Ex vivo MEA recordings

#### MEA setup

Mice were kept on a 12/12 h light/dark cycle, dark-adapted for 4–16 h before the experiment, and killed under dim red light by cervical dislocation, with or without preceding exposure to CO_2_. Experiments were performed during daylight circadian times (experiment start in the morning or early afternoon). The eye cups were removed, put in Ringer solution (in mM: 110 NaCl, 2.5 KCl, 1 CaCl_2_, 1.6 MgCl_2_, 10 d-Glucose, and 22 NaHCO_3_) bubbled with 5% CO_2_/95% O_2_. The retina was isolated and attached to a nitrocellulose filter (Millipore) with a central 2 × 2 mm hole, with the optic nerve head centered.

All recordings were performed with a perforated 60-electrode MEA (60pMEA200/30iR-Ti-gr, Multichannel Systems, Reutlingen). The electrodes are arranged on a square grid with a 200 µm distance between neighboring electrodes. Experiments were performed as described previously^50^. Briefly, the mounted retina was placed ganglion cell-side down in the recording chamber, and good electrode contact was achieved by negative pressure through the perforated MEA. The tissue was superfused with Ringer solution at 34 °C. Data were recorded at 25 kHz with a USB-MEA-system (USB-MEA1060, Multichannel Systems, Reutlingen) or an MC-Card-based MEA-system (MEA1060, Multichannel Systems).

####  Ganglion cell spike recordings

Data was high-pass filtered (500 Hz, 10th order butterworth filter), and spike waveforms and spike times were extracted from the raw data using Matlab (MathWorks). Spike sorting and thereby assignment of spikes to individual units (presumably ganglion cells) was performed semi-manually with custom written software (Matlab). Quality of each unit was individually/manually assessed by interspike interval and spike shape variation. Data analysis was based on the spiking responses of individual units. We estimated the instantaneous firing rate of ganglion cells by convolving the spike train (i.e., time series of 0’s and 1’s) with a Gaussian with sigma of 40 ms.

#### 9-*cis* retinal pharmacology

9-*cis* retinal (Sigma R5754) was dissolved in 99.96% ethanol (VWR 20821.330) for a 50 mM stock solution (Fig. 8). For the experiments, stock was diluted 1000-fold in Ringer solution, for a final concentration of 50 µM. 9-*cis* retinal Ringer solution was perfused at the beginning of the dim segments of these experiments (Fig. 8a). Given the flow rate of our perfusion system, it took about 1.5 min for the MEA chamber to be completely filled with the 9-*cis* retinal Ringer solution; after 4 min at full concentration, we switched back to normal Ringer solution. 9-*cis* retinal Ringer solution was prepared fresh from stock for each experimental perfusion event.

#### ERG recordings

Ex vivo ERG recordings were performed as described previously utilizing the same 60-electrode MEA system as described above^50^. An Ag/AgCl pellet reference electrode (Science Products E-201ML) was connected instead of the internal reference electrode of the MEA chamber. The AgCl reference was positioned 2–3 mm above the center of the MEA electrode field and was optically shielded from direct visual stimulation. Synaptic transmission from photoreceptors to bipolar cells was blocked with 50 µM L-AP4 (Sigma A7929 or Abcam ab120002), 10 µM NBQX (disodium salt, Tocris 1044), and 10 µM RS-CPP (Tocris 0173). Glial currents (slow PIII component) were inhibited with 100 µM BaCl_2_ (Sigma 342920)^51^. Data were low-pass filtered (300 Hz, 4th order Butterworth filter) and downsampled to 1 kHz. Noisy electrodes were discarded and all remaining electrodes were averaged for the analysis of ex vivo ERG responses.

### Light stimulation (ex vivo experiments)

#### Experimental control of light intensities

Visual stimulation followed the same protocol described before^52^. Briefly, the retina was stimulated with full-field gray scale visual stimuli with a computer-controlled digital light processing (DLP) projector (PG-F212X-L, Sharp or K11, Acer) and focused onto the photoreceptors through the condenser of the microscope (Supplementary Fig. 1c). The stimulus projector produced output spanning 3 log units of light intensities (i.e., 1000-fold difference between black (“0”) and white (“255”) pixels). We linearized the gamma function of the projector output. The light path contained a shutter and two motorized filter wheels with a set of neutral density (ND) filters (Thorlabs NE10B-A to NE50B-A), having optical densities from 1 (“ND1”, 10^1^-fold light attenuation) to 5 (“ND5”, 10^5^-fold light attenuation). To achieve light attenuation stronger than 5 log units, we serially combined an ND5-filter in one filter wheel with another ND filter in the second filter wheel, to achieve optical densities from 6 to 10. While changing the ND filters, we closed the shutter to prevent intermittent exposure to bright light. We usually started the experiments with ND8 (i.e., combination of ND5 and ND3 filter), and step by step increased the ambient stimulation luminance by changing the ND filters by 1 unit. Unless otherwise noted, we presented the same set of visual stimuli at each ND level during an experiment.

#### Light intensity measurements

We measured the spectral intensity profile (in µW cm^–2^ nm^–1^) of our light stimuli with a calibrated USB2000+ spectrophotometer (Ocean Optics). We then transformed the stimulus intensity into rod-effective photons cm^–2^ s^–1^ by converting the spectrum to photons cm^–2^ s^–1^ nm^–1^, and integrating it with the normalized spectrum of rod sensitivity^53^. In addition, for comparison we report stimulus intensity in equivalents of photoisomerizations per rod and second (R* rod^–1^ s^–1^), assuming dark-adapted rods, by multiplying the photon flux with the effective collection area of rods (0.5 µm^2^)^54^. The results for a stimulus intensity of “30” range from 2 × 10^8^ rod-effective photons cm^–2^ s^–1^ (1 R* rod^–1^ s^–1^, ND8) to 2 × 10^15^ photons cm^–2^ s^–1^ (10^7^ R* rod^–1^ s^–1^, ND1), see Fig. 2b, c. Note that the intensity values given as R* rod^–1^ s^–1^ (isomerizations events s^–1^ rod^–1^) serves for only comparison. It truly reflects photoisomerizations only at low intensities; at high backgrounds, bleaching adaptation leads to a much lower effective rate of isomerizations.

#### Contrast

We report stimulus contrast in “Michelson contrast” and, for comparison, also in “Weber contrast”. For a flash stimulus of intensity *I*_stim_, presented on a background of intensity *I*_back_, the definitions are as follows:

$${\mathrm{Michelson}}\,{\mathrm{contrast}} = \frac{{I_{{\mathrm{stim}}} - I_{{\mathrm{back}}}}}{{I_{{\mathrm{stim}}} + I_{{\mathrm{back}}}}}.$$

$${\mathrm{Weber}}\,{\mathrm{contrast}} = \frac{{I_{{\mathrm{stim}}} - I_{{\mathrm{back}}}}}{{I_{{\mathrm{back}}}}}.$$

### Specific stimuli and response analysis (ex vivo recordings)

#### Ganglion cell spiking responses

*Stimulus*. Ganglion cell spiking responses were probed with full-field contrast steps (step duration: 2 s) on a gray (“30” RGB pixel intensity) background (positive contrast: “30” → “50”, Michelson: + 0.25, Weber: + 0.66; negative contrast: “30” → “10”, Michelson: –0.49, Weber: –0.66, see Figs. 2b, 8b). Five positive and five negative steps were interleaved and presented as one block, and the firing rate to these five repetitions was averaged and taken as “one response”. The firing rate curves on the right in Figs. 2d, e, and 8c represent these “responses”, which were used for further analysis; the rasters on the left show the underlying five individual responses.

*Responsiveness*. Whether or not a ganglion cell responded to a block (five repetitions) of contrast steps was determined manually. For each unit and each stimulus block, we manually inspected spike raster plots and firing rates. If a cell responded clearly and consistently to at least three out of five repetitions within one stimulus block, it was considered as “responding” and was tagged with “1”. Since the purpose of this analysis was to see if rods can drive light responses in ganglion cells, also purely “negative responses” (stimulus-evoked spike suppression) was counted as a response. Stimuli for which a cell responded to only 1 or 2 repetitions or for which the response was weak and/or sluggish were tagged with “0.5”. If a cell did not respond during a stimulus block, it was tagged with “0”. The average value of these assignments across all units was used as the value for “responsiveness” in Fig. 3a, c, d.

*Amplitude*. The amplitude of the response (used in Fig. 3b) was determined automatically as follows: first, the baseline firing rate was subtracted from the response (baseline firing rate was defined as the mean firing rate during 1300 ms before contrast step onset); second, we took the absolute value of the response (such that also negative deflections in the firing rate would be recognized as a response of the cell to stimulation); third, looking at all four brightness transitions (onsets and offsets of the positive and negative contrast steps), we took the maximal response value within 50–400 ms after the contrast step. This gave one “amplitude” value for each ganglion cell and for each stimulus block. For further analysis, we only considered amplitude values during stimulus blocks to which the cell actually responded (responsiveness tags “0.5” or “1”, see above). These amplitudes were normalized for each ganglion cell separately to its maximal response across the experiment.

*Averaging across experiments*. In most experiments, full-field contrast steps were presented at the same time points after light-level transitions, with the earliest presentation about 4 min after the ND filter switch and then regularly every 5 min (Protocol 1 in Supplementary Fig. 1b; other stimuli, not discussed here, were presented in between. Note that the other stimuli were also presented on a background of “30” and their maximal intensity did not exceed “60”, ensuring no excessive contribution to light adaptation compared to the full-field contrast step stimuli.) In the experiments depicted in Fig. 3b, c, we changed the order of stimuli and presented full-field contrast steps more closely after the light level switch (Protocol 2 in Supplementary Fig. 1b). In those experiments, we probed the 10^4^ R* rod^–1^ s^–1^ and 10^5^ R* rod^–1^ s^–1^ light levels at even tighter intervals (Protocol 3) to follow the dynamic changes of ganglion cell responses with higher temporal precision. Supplementary Fig. 1b shows how the data points in Fig. 3 were averaged across experiments in which different stimulus protocols were used.

#### Ganglion cell spiking responses with 9-*cis* retinal

*Relative response strength*. For the experiments with 9-*cis* retinal (Fig. 8), we presented the stimuli at two interleaved light levels: 10^2^ R* rod^–1^ s^–1^ (10 min each), and 10^5^ R* rod^–1^ s^–1^ (30 min each). The goal was to determine the evolution of ganglion cell response strength during the 30 min at 10^5^ R* rod^–1^ s^–1^, when returning to that light level from the interleaving dimmer period. To this end, we took the average spike rate to the last 50 stimulus repetitions within a 30-min segment as a template (Fig. 8c, top), and made a least-square-error linear fit of the template to all responses along the same 30-min segment (Fig. 8c, bottom):

$$\rm response\sim m\cdot template + {\mathrm{b}}.$$

Here, we independently fitted the first 1000 ms of the responses to the four stimulus edges (i.e., 1000 ms after beginning and end of the positive and negative contrast steps). The linear term *m* of this fit was interpreted as the “relative response strength” and used for further analysis, the constant term b was not further used. Figure 8d shows the evolution of the response strength for one example ganglion cell during the one experimental segment at 10^5^ R* rod^–1^ s^–1^ shown in Fig. 8b.

*Population analysis*. We recorded from *n* = 6 *Cnga3* ^*–/–*^ retinas with this protocol. For four retinas, we applied 9-*cis* retinal Ringer during the three last experimental segments, as shown in Fig. 8a. For the other two retinas, we only recorded under control conditions. For each of the four stimulus edges, data as shown in Fig. 8d were counted as “*n* = 1” for the population analysis. We pooled this data for all experimental segments, for all ganglion cells, and across all six experiments, separately for the “control” and “retinal” experimental segments. Because this approach is based on template matching, we only included experimental segments in the population data during which ganglion cell spiking was robust, and in which there was a template to be matched (e.g., if the response of a cell consists of complete suppression of spiking, this will not yield a template that can be matched). Whether or not to include the responses of a ganglion cell during an experimental segment in the analysis was judged manually and independently for each of the four stimulus edges. As a result, the number of included experimental segments (“*n*”) varies across the different experimental conditions (Fig. 8e).

*Statistical analysis*. For each time point after returning to 10^5^ R* rod^–1^ s^–1^, we compared the relative response strength in control and retinal conditions with a permutation test. Here, we combined all values (control and retinal), and randomly sampled *n* values, with *n* being the smaller of the two population sizes (which was the “retinal” population in each case). We then split this sample in two, measuring the distance between their medians. We repeated this 10^5^ times. Coming from the same population, the distribution of these distances is symmetric around 0. The null hypothesis was that the experimentally observed distance between the medians of control and retinal conditions was from the same distribution, with the alternate being that median of retinal conditions is smaller than that of control. We took as *p*-value to support the null hypothesis the fraction of samples (out of 10^5^) for which the distance between medians was at least as large as the experimentally observed value. The *p*-value is color coded in Fig. 8, with *p* < 0.01 shown in green, *p* < 0.05 in yellow, and *p* ≥ 0.05 in white.

#### ERG recordings

*Stimulus*. For ex vivo ERG recordings, we used a series of 50 ms flashes of different positive contrasts (Fig. 1a). One stimulus set consisted of four flashes with Michelson contrast +0.79 (Weber: +7.44, “30” → “255”), and two flashes each of Michelson contrast +0.6 (Weber: +2.97, “30” → “120”), +0.33 (Weber: +0.99, “30” → “60”), and +0.99 (Weber: +999, “0” → “255”). In order to achieve the high contrast (+0.99), it was necessary to intermittently reduce the gray background from “30” to “0”. About 16 such stimulus sets were shown among other stimuli at each light level (30 min) from 10^0^ R* rod^–1^ s^–1^ to 10^6^ R* rod^–1^ s^–1^. The other stimuli, not discussed here, were limited to a brightness range between “0” and “60”, presented on a background of “30”.

*Analysis*. We quantified the strength of the recorded ERG signal by measuring the mean amplitude of the negative voltage deflection during 300 ms directly after the onset of a 50 ms flash. The 300 ms voltage signal preceding the flash was used as a baseline to test for significance of the flash-elicited responses (Wilcoxon rank sum test). Significance testing was performed by using flashes of three consecutive stimulus sets, i.e., *n* = 12 flashes for contrast +0.79 and *n* = 6 flashes for the other contrasts. Figure 1b shows the moving average for this analysis (averaging three stimulus sets per data point, shifting by one stimulus set for the next data point; no averaging was done across light level transitions).

*Response reliability*. ERG responses at high light levels were usually very small, but nevertheless often clearly distinct from the voltage fluctuations of the background activity. As a measure of the reliability of such small signals, we devised a “response reliability index”, which we calculated from the statistical measure of the presence of a response (namely *p*-value resulting from the Wilcoxon rank sum test, see above) according to the relationship depicted in Supplementary Fig. 1a.

### In vivo dLGN recordings

#### In vivo setup

Mice were anesthetised using a single dose of urethane (30% w/v in dH_2_O, 1.6 mg kg^−1^, i.p) and placed in a stereotaxic frame (SR 5-M; Narishige, Japan) on a temperature-regulating 37 °C heat mat (Harvard Apparatus, UK). A craniotomy was drilled above the coordinates for the dLGN (B–2.2–2.6 mm, ML 1.5–3 mm) relative to the mouse stereotaxic atlas^55^. A 32-contact recording electrode (A4 × 8–5 mm-50-200-177/413-A32; Neuronexus, USA) was lowered into the dLGN and extracellular spiking activity collected through a Recorder64 system (Plexon, USA). Light stimuli were delivered to the eye contralateral to the recorded brain hemisphere. Upon completion, animals were killed by cervical dislocation and the brain fixed in 4% paraformaldehyde. Electrode placement (the electrode was dipped in fluorescent dye; CM-Dil, Life Technologies, UK) was verified in post hoc histology.

#### Light stimuli for Cnga3^−/−^ mice

For in vivo experiments presented in Fig. 4, we delivered multispectral stimuli using a Spectra X light engine (Lumencor, USA). Stimuli were created by stepping four LEDs in combination from a low background to a high level (blue, cyan, green, and yellow, *λ*_max_ = 430 nm, 480 nm, 511 nm, and 575 nm, respectively). Light stimuli were presented through a light guide to the atropine-dilated eye as diffuse illumination of a Lambertian disc (10 mm in diameter, placed <5 mm from corneal surface). A circular ND wedge (100FS04DV.4, Newport) in the light path between the exit point of the light engine and the end of the optical fiber allowed light intensity to be modulated over a 4 log unit range.

For contrast response assessments in *Cnga3* ^*–/–*^ mice (Fig. 5), multispectral stimuli were presented using a system constructed in-house (components from Thorlabs and Edmund optics), comprising three LEDs (*λ*_max_ = 405, 470, and 617 nm). Stimuli here were delivered to the subject by a 7 mm diameter light guide (placed ~5 mm from the corneal surface and enclosed within a spectrally neutral reflective cone to provide diffuse full-field illumination) and intensity was adjusted using an ND filter wheel.

In all cases, spectral power densities for each LED were measured using a calibrated spectroradiometer (Bentham Instruments Ltd., UK). These were converted to retinal irradiance in rod-effective photon cm^–2^ s^–1^ by converting the corneal irradiance and correcting for the pre-receptoral filtering of the lens. The background intensity at the brightest light levels used in vivo was 4.52 × 10^14^ rod-effective photon cm^–2^ s^–1^ (10^6.35^ R* rod^–1^ s^–1^) for the experiments shown in Fig. 4, and 1.63 × 10^14^ rod-effective photon cm^–2^ s^–1^ (10^5.9^ R* rod^–1^ s^–1^) for Fig. 5.

For experiments in Fig. 4, 1200 flashes (duration: 50 ms) were shown at 1 Hz at each light level (+0.75 Michelson contrast for rods). These flashes were interleaved with a weaker contrast (+0.5, data not analyzed here), thus the protocol took 40 min per light level. For contrast sensitivity experiments (Fig. 5), we uniformly modulated background light intensity (square-wave 0.25 Hz) at a range of contrasts (20–96%) in eight interleaved blocks of six stimulus repeats. We also included spectral modulations designed to evoke varying degrees of rod contrast (not included in the analysis presented here) such that each block of stimuli took 6 min. Stimulus presentation started 30 s after stepping to the tested background.

#### Data analysis in *Cnga*^*−*/−^ mice

For experiments with fixed contrast (Fig. 4), we measured 40 light-responsive multiunits from three mice. Four multiunits were excluded because they stopped responding completely after the first light level switch. In one mouse, recordings could only be performed up to the highest light level of 10^6.35^ R* rod^–1^ s^–1^, but not for the last mesopic (10^3.35^ R* rod^–1^ s^–1^) repetition. Firing rate has been calculated by convolving the spike train (i.e., time series of 0’s and 1’s) with a Gaussian with sigma of 5 ms. Then, responses to 10 flashes were averaged (=1 group). For each group, we calculated the mean background firing rate for the 190 ms directly before stimulus onset. The background firing rates from 20 groups was then averaged and taken as the mean background firing rate for these 20 groups. The mean response rate 50–250 ms after the flash stimulus was considered as response. We applied a Wilcoxon rank sum test (1-sided) to test for significant differences between the 20 background and the 20 response values, i.e., we tested for significant light responses. These significance tests were performed on a running average with shifts of two groups for each data point. No averaging was performed across light-level borders. This resulted in ~350 *p*-values per recorded multiunit over the whole series of light levels. The measured *p*-values were then transformed into a response reliability index as shown in Supplementary Fig. 1a.

For contrast sensitivity experiments (Fig. 5), we measured responses from 86 LGN multiunits in four mice. Responses at each contrast level and light level were combined to form peri-event histograms (40 ms bin size smoothed with a 5 bin boxcar filter) across four 12 min time bins, each comprising two blocks of stimuli. For analysis, the first trial from each block was excluded such that each bin contained the average of 10 stimulus repeats where intensity varied symmetrically around the background. Response amplitude was defined as the peak-to-trough variation in firing rate under each condition. Contrast response relationships for each channel were then calculated from the average of all LGN multiunits and fitted with a 4-parameters sigmoid function (Graphpad prism).

#### Detecting rod contributions to the visual response of Opnmw^R^;Opn4^−/−^ mice

*Stimulus*. We presented a series of blue, cyan, and red flashes (50 ms duration) at 2 Hz frequency on a light adapting violet background (*λ*_max_ = 400 nm, Fig. 6a). We used the Spectra X light engine (Lumencor, Beaverton, OR, USA), the circular ND wedge described in section “Light stimuli for *Cnga3* ^*−/−*^ mice”. Flashes followed a pseudorandom order for colors (*λ*_max_ = 430, 480, and 630 nm respectively for blue, cyan, and red LEDs) and intensities (15 different levels per color) to prevent contrast adaptation in the response.

*Stimulus intensity and contrast*. Our estimate of S-, L-cone, and rod normalized sensitivity for calculating flash contrasts was based upon Govardovskii nomograms^8^, using *λ*_max_ = 365 nm for S-cones, *λ*_max_ = 556 nm for L-cones^17^, and *λ*_max_ = 498 nm for rods^11^, adjusted for photopigment optical density (POD)^56^ and lens absorption, using a function adapted from^57^:

$${\mathrm{Normalized}}\,{\mathrm{sensitivity}} = 10^ {- {\mu(\lambda )D}} \times {\mathrm{sensitivity}}/{\mathrm{max}}\left( {{\mathrm{sensitivity}}} \right),$$

with sensitivity = (1 − 10^−POD×S(λ)^), S(λ) is the pigment nomogram, *D* is the lens thickness (*D* = 2.07 mm)^57^, and *μ*(λ) is the attenuation coefficient calculated as

$$\mu \left( \lambda \right) = c\left( {\lambda ^d - \lambda _0^d} \right)\,{\mathrm{for}}\,\lambda < \, \lambda _0^d;\mu \left( \lambda \right) = 1\,{\mathrm{otherwise}}{\mathrm{.}}$$

The values for *c* and *λ*_0_ (=wavelength of maximal lens transmission) were obtained by fitting tabulated data (*c* = 5.33 × 10^4^; *d* = −2.27; *λ*_0_ = 700 nm)^58^. The absolute stimulus intensity of the violet background (effective photons cm^–2^ s^–1^ flux for the brightest background: rod-opsin 1.3758 × 10^13^ (≈ 10^4.84^ R* rod^–1^ s^–1^; in Fig. 6, this condition is tagged with “10^5^ R* rod^–1^ s^–1^”), L-opsin: 1.3473 × 10^13^, S-opsin: 1.5833 × 10^13^), and contrast of the flashes (assuming POD = 0.1 for cones, POD = 0.01 for rods, and *c* = 5.33 × 10^4^) are depicted in Fig. 6b.

*Analysis*. About 45 Peri-stimulus time histograms (PSTHs) were estimated (15 intensities × 3 colors) for each light-responsive unit and a PSTH matrix with mean firing rate responses (<*fr*>) was generated. In order to remove the high-frequency noise due to the finite number of trials, we computed the eigenvalue decomposition of the PSTH covariance matrix (PSTH^T^×PSTH). Then we selected the smallest subset of eigenvectors whose associated eigenvalues accounted for >90% power of the PSTH covariance matrix. Finally, we used the selected eigenvectors and their projections to reconstruct a “de-noised” version of the original PSTH matrix. The 45 responses were then calculated as the Euclidean norms of the “de-noised” PSTHs as follows:

$${\mathrm{Response}} = \sqrt {{\sum}{\left \langle {fr} \right \rangle^2}} ,$$

with the summation taken across 20 time bins (time bin duration 15 ms) in the first 300 ms after the flash onset. We initially evaluated the possibility to measure flash responses as increments/decrements in firing rate in respect to the baseline. However, we chose to use the Euclidean norm because we observed that a significant fraction of units exhibited multiphasic responses where those increments and decrements in firing rate tended to cancel each other out.*Statistical analysis of colored flash responses*. The procedure for statistical analysis and their results are described in Supplementary Table 1.

### Computational model

For the sinusoidal stimulus used in the computational model (Fig. 7a), we report the Michelson contrast based on the minimum and maximum deflections of the sinusoid:

$${\mathrm{Michelson}}\,{\mathrm{contrast}}_{{\mathrm{sinusoid}}} = \frac{{I_{{\mathrm{max}}} - I_{{\mathrm{min}}}}}{{I_{{\mathrm{max}}} + I_{{\mathrm{min}}}}}.$$

We have employed the model of Invergo et al.^20^ (BioModels BIOMD0000000578) to simulate the phototransduction cascade within the rod outer segment. This model is an adaptation to mouse rods of previous models intended to simulate the phototransduction cascade in amphibians^59, 60^. The current model of Invergo et al. describes the phototransduction cascade on the system level: based on a reaction network for the molecular species, a system of ODE is derived by simplifying assumptions like mass action kinetics. The numerical solution of this ODE system yields the time dependence of each of the involved molecular species and as the main outcome the photoresponse to a prescribed stimulus. We have implemented the model in the simulation software COPASI^61^. The original parameters of this model had been fit to biochemical and physiological data based on very different stimuli than the stimulus used in our study, namely to very brief and moderate-intensity flash stimuli on a dark-adapted rod (lasting 10s of milliseconds of at most 2000 R* rod^–1^ flash^–1^). Compared to the original parameters of Invergo et al., we have set the total number of rhodopsin molecules (*Rhod*_total_) to 2.6 × 10^7^ (Fig. 7a–e, corresponding to the left column in Supplementary Fig. 4) instead of 10^8^, and varied this parameter up to the upper bound reported for rods^62^ (7 × 10^7^, right column in Supplementary Fig. 4). We set the parameter *k*_Ops_ (the efficiency of opsin binding to transducin^33, 34^) to 10^−9^ instead of 10^−13^, which resulted in a more pronounced saturation (the dependency of the model behavior on this parameter is shown in Supplementary Fig. 4). We have varied the parameter for the rhodopsin regeneration rate (*k*_Rrecyc_) to try and mimic the different experimental conditions (ex vivo, in vivo) and to investigate the dependency of rod responses on that parameter (rows in Supplementary Fig. 4). Given the long duration and high-intensity range of our stimulus, we took into account that arrestin, transducing, and recoverin translocate between the outer and inner segments^21, 22^, resulting in a near-exchange of these molecular species between inner and outer segment (Fig. 7c). Under intense illumination, arrestin translocates from the inner segment to the outer segment, while transducin and recoverin move in the opposite direction. In the outer segment, it has been proposed that the sequestering of arrestin into a “storage” oligomeric state, and functional monomeric state, may be important for achieving the right functional concentration^59^. As in the original model by Invergo et al., we have included arrestin oligomerization and therefore allow for the presence of arrestin transport and arrestin oligomerization. We have observed that the reorganization of oligomers is so fast that the effect will be negligible in influencing the time course of response recovery studied here. But even then, even a small variation due to the interaction between arrestin transport and arrestin oligomerization is reflected in the resulting photoresponse. We have implemented the translocation events with their own dynamic properties (solid lines in Fig. 7c–e; Supplementary Fig. 4), as a first-order reaction, with the reaction constant being proportional to the difference between the current total concentration of the species in the outer segment, and the desired concentration. By this way, the concentration of the corresponding species was smoothly driven to the desired level. The reaction constant itself was adjusted so that the final concentration would be reached with a time course described in the original publications^21, 22^. Alternatively, we implemented translocation as a simplified and artificial stepwise change of concentration upon light-level transitions (dashed lines in Fig. 7c, model behavior in Fig. 7e). None of our parameter adjustments changed the model behavior to the original stimuli used for parameter fitting by Invergo et al.^20^ Our model is available in BioModels^63^ under the identifier MODEL1710030000.

### Data availability

The experimental data that support the findings of this study are available from the authors on reasonable request. Raw measurements from ERG recordings (Fig. 1; Supplementary Fig. 2) are available on Figshare (10.6084/m9.figshare.5492626). The computational model is available in BioModels^63^ under the identifier MODEL1710030000.

## Electronic supplementary material

Supplementary Information
