# Supplementary material for: Rods progressively escape saturation to drive visual responses in daylight conditions
Source: Nat Commun. 2017 Nov 27;8:1813. doi: 10.1038/s41467-017-01816-6 (PMC5703729; doi:10.1038/s41467-017-01816-6)
Supplement: Supplementary file 1 — Supplementary Information [file 41467_2017_1816_MOESM1_ESM.pdf]

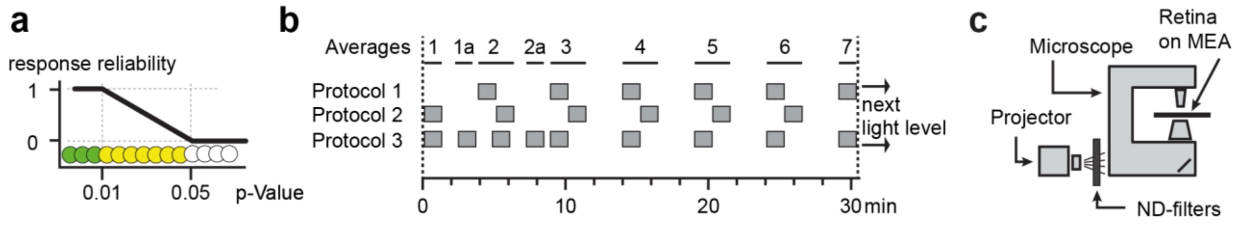

Supplementary Figure 1. Supplementary information about methods.

a Calculation of response reliability (see Figs. 1, 4 and Supplementary Fig. 2) was based on the significance (p-Value) of an observed response. Responses which hardly differed from background activity ($p>0.05$, color-coded with white disks in Figs. 1b, c and 4b) were considered unreliable (reliability = 0). Correspondingly, highly significant responses ($p<0.01$, color-coded with green disks) were considered very reliable (reliability = 1). p-values between 0.05 and 0.01 (color-coded yellow) were converted linearly to reliability-values between 0 and 1.
b Full-field contrast steps (see Fig. 2a) were always presented in blocks of 5 repetitions, lasting ca. 1 min (gray rectangles). In different experiments, we presented these blocks at different times after transitioning to a new light level. In most experiments we used Protocol 1, in which the first presentation started at time 4 min. In some experiments, we used Protocol 2 instead. We averaged across these different experiments as indicated on top, yielding 7 data points at each light level (see Fig. 3). At the light levels causing 10^4 R* rod^{-1} s^{-1} and 10^5 R* rod^{-1} s^{-1}, we used Protocol 3 instead of Protocol 2, yielding the additional data points 1a and 2a.
c Schematics of the experimental setup for *ex vivo* experiments

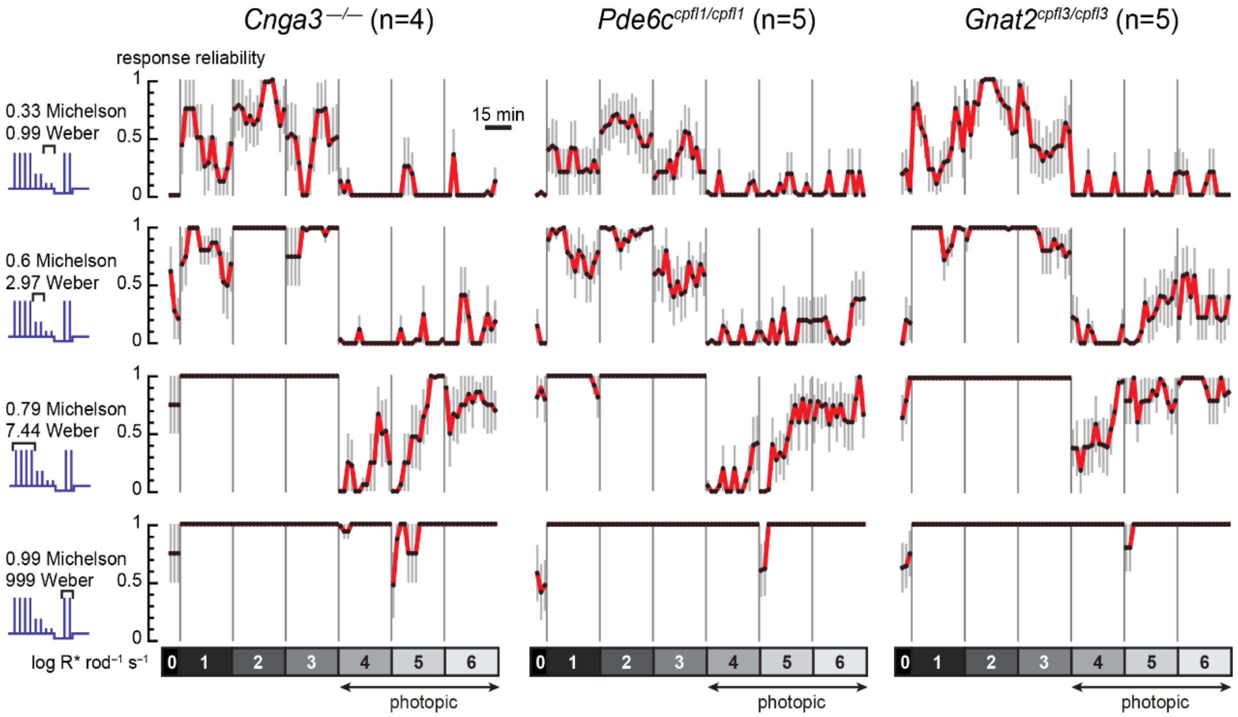

Supplementary Figure 2. ***Ex vivo*** **ERG recordings from isolated Cnga3$^{-/-}$, Pde6c$^{cpfl1/cpfl1}$, and Gnat2$^{cpfl3/cpfl3}$ retina.**

Response reliability of ERG responses (mean ± s.e.m.) for *Cnga3*$^{-/-}$ (n=4 retinas), *Pde6c*$^{cpfl1/cpfl1}$ (n=5), and *Gnat2*$^{cpfl3/cpfl3}$ (n=5) retinas to flashes of 0.33, 0.6, 0.79, and 0.99 Michelson contrast. Response reliability was calculated from the p-Values resulting from comparing background and response activity (as described in Supplementary. Fig. 1a and Methods). Similar contrast dependence of ERG responses was found for all three mouse strains. At high light levels (10^4 R* rod^{-1} s^{-1} to 10^6 R* rod^{-1} s^{-1}), responses could be detected reliably for stimuli with Michelson contrast of 0.79 and higher (for *Gnat2*$^{cpfl3/cpfl3}$ already for stimuli with contrast 0.6). For weaker contrast, only responses to low and medium light levels (10^0 R* rod^{-1} s^{-1} to 10^3 R* rod^{-1} s^{-1}) were reliably detectable. The time-dependent re-emergence of rod-driven light responses found for stimuli of contrast 0.79 in *Cnga3*$^{-/-}$ retinas was also present in the two other mouse strains.
Raw data underlying this plot are available on Figshare (doi.org/10.6084/m9.figshare.5492626).

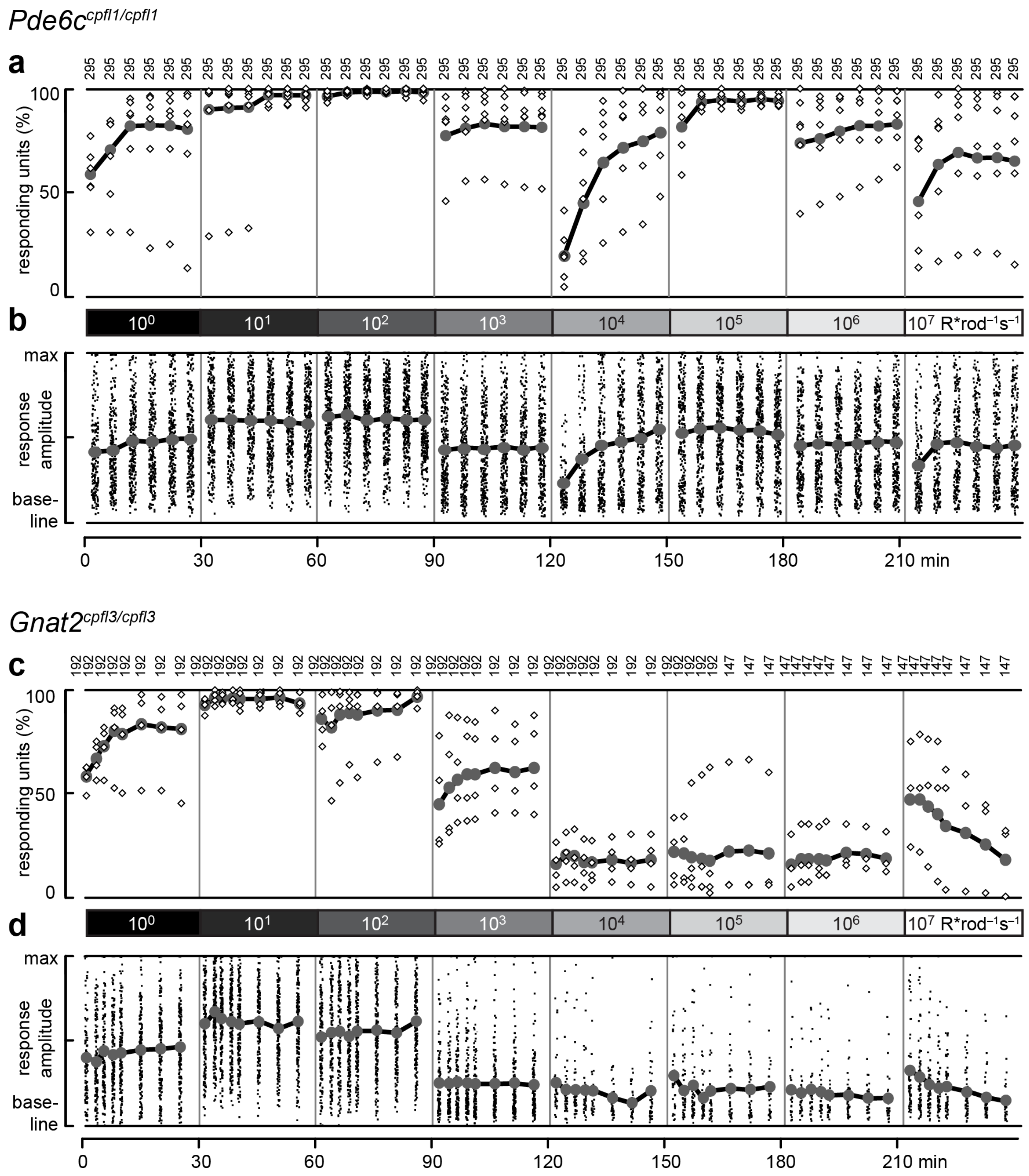

Supplementary Figure 3. Responsiveness of ganglion cells in isolated *Pde6c*$^{cpfl1/cpfl1}$, and *Gnat2*$^{cpfl3/cpfl3}$ retina.

a, b Percentages (**a**) and relative amplitudes (**b**) of responding units in *Pde6c*$^{cpfl1/cpfl1}$ retinas (n=5). The percentage of responding units dropped in the beginning of high light levels, but recovered with a similar time course as found in *Cnga3*$^{-/-}$ retinas (Fig. 3a). On a population level, the relative amplitude was stable across light levels, except for a small drop in the beginning of 10^4 R* rod^{-1} s^{-1}, comparable to our findings in *Cnga3*$^{-/-}$ (Fig. 3d).
c, d Percentages (**c**) and relative amplitudes (**d**) of responding units in *Gnat2*$^{cpfl3/cpfl3}$ retinas (n=4). Consistent with our findings in *Pde6c*$^{cpfl1/cpfl1}$ and *Cnga3*$^{-/-}$ retinas, rods drove visual responses at any light level also in *Gnat2*$^{cpfl3/cpfl3}$ retinas.

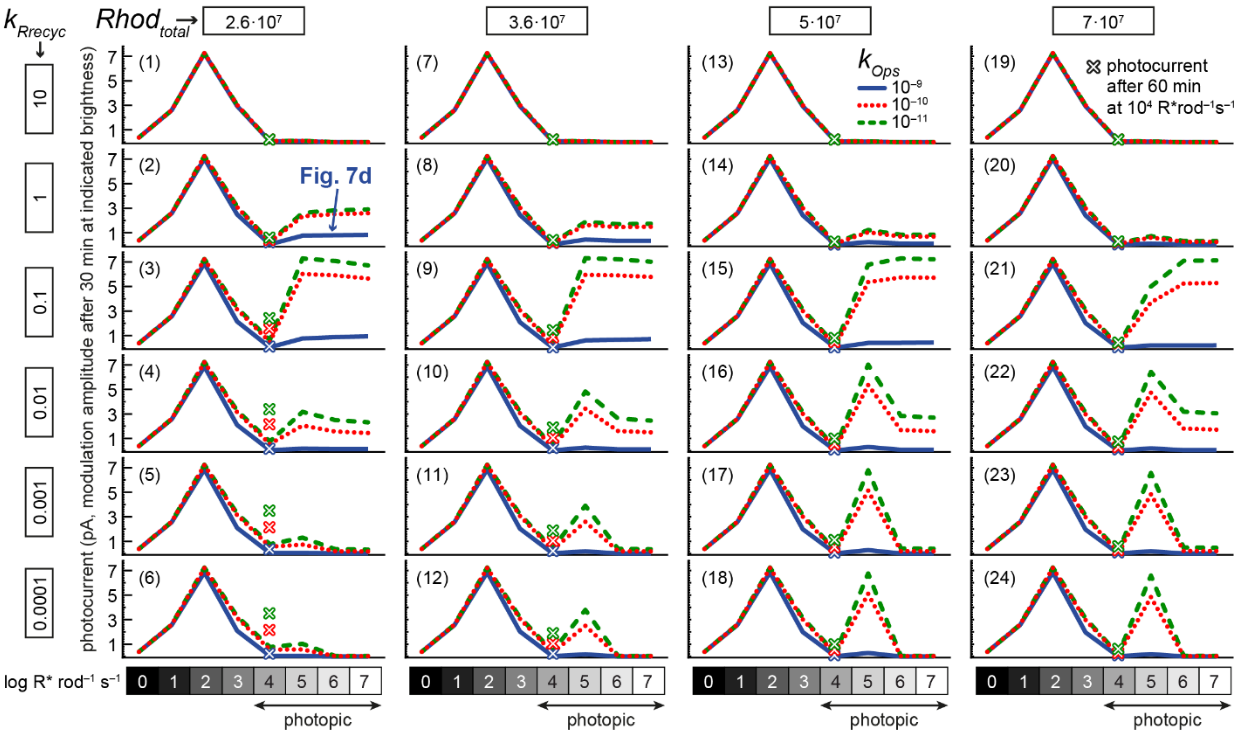

Supplementary Figure 4. Parameter variation in the computational model.

Amplitude of photocurrent modulation (min-to-max, to moderate contrast sinusoidal stimulus) at the end of 30 min exposure to the 8 different background light levels (crosses: after 60 min of exposure to 10^4 R* rod^{-1} s^{-1}, the photopic light level with the slowest adaptation rate), for different parameter combinations. Columns: different total rhodopsin concentration ($Rhod_{total}$). Rows: Different rhodopsin regeneration rate (k_{Rrecyc}), as multiple of the standard rate used in Fig. 7a-e. Differently colored traces: different values of k_{Ops}, the rate with which opsin binds transducin to activate the cascade. All other model parameters are as in Fig. 7d (the blue curve in panel 2 reflects the data in Fig. 7d).

Supplementary Table 1. Statistical analysis of responses to blue, cyan and red flashes in the dLGN of Opn1mw^R:Opn4$^{-/-}$ mice (Fig. 6).

a $POD_{L\text{-}opsin} = 0.1$; $c = 5.33*10^4$

	R^2_{BCR}	R^2_{null}	ΔR^2
**10^3 R*/rod/s**	47.5%	40.2%	7.3%
**10^4 R*/rod/s**	48.7%	48.3%	0.4%
**10^5 R*/rod/s**	57.3%	52.5%	4.8%

b varying $POD_{L\text{-}opsin}$ and c

**10^3 R*/rod/s**	R^2_{BCR}	R^2_{null}	ΔR^2
0.75*c	47.5%	40.6%	6.9%
1.25*c	47.4%	39.9%	7.6%
$POD_{L\text{-}opsin} = 0.01$	47.5%	40.4%	7.1%
$POD_{L\text{-}opsin} = 1$	47.5%	38.6%	8.9%

**10^4 R*/rod/s**	R^2_{BCR}	R^2_{null}	ΔR^2
0.75*c	48.7%	48.3%	0.4%
1.25*c	48.7%	48.3%	0.4%
$POD_{L\text{-}opsin} = 0.01$	48.7%	48.4%	0.3%
$POD_{L\text{-}opsin} = 1$	48.7%	47.4%	1.3%

**10^5 R*/rod/s**	R^2_{BCR}	R^2_{null}	ΔR^2
0.75*c	57.3%	52.0%	5.3%
1.25*c	57.3%	52.9%	4.4%
$POD_{L\text{-}opsin} = 0.01$	57.3%	52.5%	4.8%
$POD_{L\text{-}opsin} = 1$	57.3%	51.8%	5.5%

To analyze population responses, we first normalized single unit responses by their mean value across flashes and colors. This was important to account for the variability in firing rates (both spontaneous and evoked) across units. Then, at each light level, we pooled normalized responses across units and flash intensities into three groups based on flash color (B: blue, C: cyan and R: red). We observed that, separately, each group's response to flashes (as a function of L-opsin contrast) could be well fitted by a quadratic polynomial model (i.e. two covariates and a constant). We then asked if responses to flashes of the three wavelengths, could also be adequately described by a single function. Because of the large sample size (over 1000 responses per group) comparisons based on unstandardized p-values made little sense as even the slightest difference would result in highly significant comparisons (see ref. 1 for a review). Instead, we analyzed the explained variance as a meaningful measure of the effect size. Our null hypothesis was that L-opsin contrast could account for all the explainable variance. Therefore, if L-opsin contrast was the only drive for the observed responses, we would expect that, by pooling data from all three wavelengths, the explained variance would not be smaller than the one obtained by using a more complicated model that accounted for the difference in flash colors (i.e. six covariates and a constant). To compare the explained variance under the null and alternative hypothesis we used the R^2-adjusted as in ref. 2, with the variance explained by null "pooled" model indicated as R^2_{null} and by the alternative model indicated as R^2_{BCR}. The effect size was also evaluated as $\Delta R^2 = R^2_{BCR} - R^2_{null}$.

a Results of this analysis when cone contrast was calculated using our default estimates of pigment optical density (POD) and lens correction (parameter c, see methods). The Null hypothesis can explain the variance adequately only at 10^4 R* rod^{-1} s^{-1} ($\Delta R^2 < 1\%$), but not at 10^3 R* rod^{-1} s^{-1} or 10^5 R* rod^{-1} s^{-1}.

b Impact of varying c and POD on these fits: results are stable in spite of large variations in these parameters and, again, the null hypothesis adequately describes our observations only at 10^4 R* rod^{-1} s^{-1}.

Supplementary References

1. Nakagawa, S. & Cuthill, I.C. Effect size, confidence interval and statistical significance: a practical guide for biologists. Biological reviews of the Cambridge Philosophical Society 82, 591-605 (2007).
2. Montgomery, D.C. & Runger, G.C. Applied Statistics and Probability for Engineers (John Wiley & Sons, 2010).
